# A phenotypic rescue approach identifies lineage regionalization defects in a mouse model of DiGeorge syndrome

**DOI:** 10.1242/dmm.049415

**Published:** 2022-09-27

**Authors:** Gabriella Lania, Monica Franzese, Noritaka Adachi, Marchesa Bilio, Gemma Flore, Annalaura Russo, Erika D'Agostino, Claudia Angelini, Robert G. Kelly, Antonio Baldini

**Affiliations:** ^1^ Institute of Genetics and Biophysics, National Research Council (CNR), Naples 80131, Italy; ^2^ Istituto di Ricerca e Cura a Carattere Scientifico (IRCCS) SYNLAB SDN, Via Gianturco 113, 80143 Naples, Italy; ^3^ Istituto per le Applicazione del Calcolo, National Research Council (CNR), Naples 80131, Italy; ^4^ Aix-Marseille Université, Centre National de la Recherche Scientifique (CNRS) UMR 7288, Institut de Biologie du Développement de Marseille (IBDM), Marseille 13288, France; ^5^ Department of Molecular Medicine and Medical Biotechnology, University Federico II, Naples 80131, Italy

**Keywords:** TBX1, DiGeorge syndrome, Pharyngeal apparatus, Cardiopharyngeal mesoderm, Phenotypic rescue

## Abstract

*TBX1* is a key regulator of pharyngeal apparatus (PhAp) development. Vitamin B12 (vB12) treatment partially rescues aortic arch patterning defects of *Tbx1*^+/−^ embryos. Here, we show that it also improves cardiac outflow tract septation and branchiomeric muscle anomalies of *Tbx1* hypomorphic mutants. At the molecular level, *in vivo* vB12 treatment enabled us to identify genes that were dysregulated by *Tbx1* haploinsufficiency and rescued by treatment. We found that SNAI2, also known as SLUG, encoded by the rescued gene *Snai2*, identified a population of mesodermal cells that was partially overlapping with, but distinct from, ISL1^+^ and TBX1^+^ populations. In addition, SNAI2^+^ cells were mislocalized and had a greater tendency to aggregate in *Tbx1^+/−^* and *Tbx1^−/−^* embryos, and vB12 treatment restored cellular distribution. Adjacent neural crest-derived mesenchymal cells, which do not express TBX1, were also affected, showing enhanced segregation from cardiopharyngeal mesodermal cells. We propose that TBX1 regulates cell distribution in the core mesoderm and the arrangement of multiple lineages within the PhAp.

## Teaser

**Summary:** *Tbx1* mutations model DiGeorge syndrome in mice. A phenotypic rescue approach revealed that *Tbx1* gene dosage reduction is associated with mislocalization of cell lineages contributing to the pharyngeal apparatus.

## INTRODUCTION

The embryonic pharyngeal apparatus (PhAp) is a developmental system that provides progenitors and instructions to multiple organs and tissues, including, but not limited to, the craniofacial and mediastinic muscles and bones, most of the heart, and glands such as the thymus, parathyroids and thyroid. Developmental anomalies of the PhAp underlie numerous birth defects, highlighting its developmental and genetic complexity. A textbook example of PhAp maldevelopment is DiGeorge syndrome, the most common genetic cause of which is a heterozygous deletion of a chromosomal region within 22q11.2 (in which case the clinical presentation is more complex and is designated as 22q11.2 deletion syndrome), and it can also be caused by point mutations of the *TBX1* gene (Haddad et al., 2019; Paylor et al., 2006; Xu et al., 2014; Yagi et al., 2003; Zweier et al., 2007).

The development of the PhAp depends upon the contribution of tissues derived from all three germ layers: surface ectoderm, pharyngeal endoderm, neural crest-derived cells (NCCs) and the cardiopharyngeal mesoderm (CPM). The latter contributes to a broad range of tissues and structures within the mediastinum, face and neck (Adachi et al., 2020). In the mouse, the CPM is well represented by the expression domains of the *Tbx1^Cre^* and the *Mef2c-AHF-Cre* drivers (Adachi et al., 2020; Huynh et al., 2007; Verzi et al., 2005). PhAp lineages have distinct origins and transcriptional profiles (Swedlund and Lescroart, 2020; Wang et al., 2019), and develop in close proximity or direct contact with each other, and their regionalization within the PhAp is mostly conserved across vertebrate evolution (Graham, 2001). However, the molecular code that governs regionalization has not been dissected in detail, although interactions between lineages are the subject of intense research (Calmont et al., 2009; Huang et al., 1998; Kodo et al., 2017; Mao et al., 2021; Sato et al., 2011; Shone and Graham, 2014; Warkala et al., 2020).

Loss of function of the *Tbx1* gene in the mouse has profound and broad effects on the development of the PhAp (Jerome and Papaioannou, 2001; Lindsay et al., 2001; Merscher et al., 2001), and affects the expression of thousands of genes (Fulcoli et al., 2016; Ivins et al., 2005; Liao et al., 2008; Nomaru et al., 2021; Pane et al., 2012), making it difficult to identify the effectors/targets that are critical for specific developmental functions. Phenotypic rescue strategies represent an alternative approach to focus on genes associated with phenotypic improvement.

In a search for drugs that rebalance *Tbx1* haploinsufficiency, we showed that high doses of vitamin B12 (vB12) rescued part of the mutant phenotype *in vivo* (Lania et al., 2016). Here, we show that the rescuing capacity of the drug extends to CPM-derived structures, such as the cardiac outflow tract and craniofacial muscles. Then, as a proof of principle of the usefulness of phenotypic rescue to provide insights into pathogenetic mechanisms, we leveraged vB12 treatment to identify genes and pathways that are critical for the expressivity of the rescued phenotype. This exposed a novel *Tbx1* mutant phenotype through the identification of a SNAI2^+^ subpopulation of CPM cells. Specifically, we found that, in *Tbx1* homozygous mutants, SNAI2^+^ cells were segregated from the NCCs rather than intermingled with them, suggesting a cell sorting defect. This abnormality was also evident in *Tbx1* heterozygous mutants, albeit at a reduced expressivity. Thus, in the PhAp, TBX1 dosage is important, cell autonomously and non-autonomously, for the regionalization of cell lineages. We propose that this TBX1-dependent function is part of the pathogenetic mechanism leading to severe abnormalities of the PhAp in the mouse mutants as well as in DiGeorge syndrome.

## RESULTS

### vB12 reduces the severity of the intracardiac and craniofacial phenotypes in a hypomorphic *Tbx1* mutant model

**Fig. 1.**
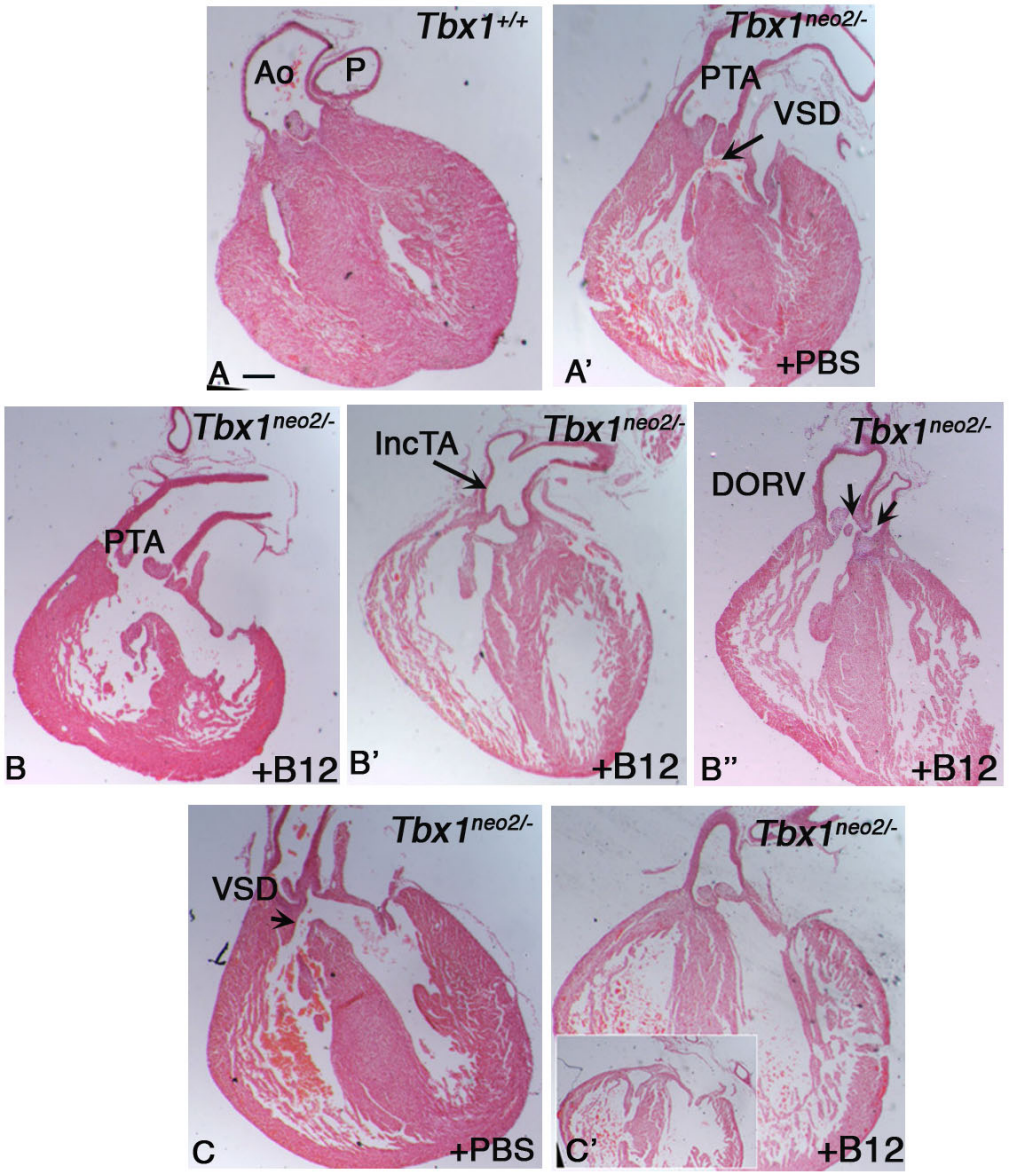
**Vitamin B12 (vB12) ameliorates cardiac outflow tract anomalies observed in *Tbx1^neo2/−^* mouse embryos.** Representative images of histological sections (coronal) of heart from *Tbx1^+/+^* and *Tbx1^neo2/−^* embryos at E18.5, treated with vB12 or PBS. (A,A′) Histological sections of hearts from *Tbx1^+/+^*+PBS (A) and *Tbx1^neo2/−^*+PBS (A′) embryos. Aorta (Ao) and pulmonary trunk (P) are separated. (B-B″) Histological sections of hearts from *Tbx1^neo2/−^*+vB12 embryos. (C,C′) Histological sections of hearts from *Tbx1^neo2/−^*+PBS (C) and vB12-treated (C′) embryos. DORV, double outlet right ventricle; IncTA, incomplete truncus arteriosus; PTA, persistent truncus arteriosus; VSD, ventricular septal defect. Scale bar: 200 µm.

High dosage of vB12 reduced the penetrance of the aortic arch phenotype and rebalanced *Tbx1* expression in haploinsufficient mice (Lania et al., 2016). However, *Tbx1*^+/−^ embryos rarely show second heart field (SHF)-related abnormalities such as outflow tract defects, which are commonly found in embryos that express low levels of *Tbx1* (Liao et al., 2004; Zhang and Baldini, 2008) or in *Tbx1*^−/−^ embryos (Jerome and Papaioannou, 2001; Lindsay et al., 2001; Merscher et al., 2001). We asked whether vB12 treatment could modify the SHF-dependent phenotype on a *Tbx1* reduced-dosage model. To this end, we exploited a hypomorphic *Tbx1* allele (*Tbx1^neo2^*) (Zhang et al., 2006) that has a loxP-flanked neomycin resistance gene inserted into an intron. *Tbx1^neo2^*^/−^ embryos exhibit heart defects similar to but less severe than those of null embryos (Zhang and Baldini, 2008). We crossed *Tbx1^neo2^*^/+^ and *Tbx1*^+/−^ mice, and injected pregnant females daily from embryonic day (E)7.5 to E11.5 with vB12 (intraperitoneal injection, 20 mg/kg/day) or vehicle (PBS, controls). Embryos were harvested and dissected at E15.5 and E18.5. Table 1 summarizes the phenotyping results. We examined 21 *Tbx1^neo2^*^/−^ embryos at E18.5 (ten controls and 11 treated with vB12). All control PBS-injected *Tbx1^neo2^*^/−^ embryos had persistent truncus arteriosus (PTA) and ventricular septal defects (VSDs) (Fig. 1A,A′). In contrast, only two of the 11 vB12-treated *Tbx1^neo2^*^/−^ embryos exhibited typical PTA (Fig. 1B), while four had an incomplete PTA, in which there was an unseptated valve but a distal separation of the aorta and pulmonary trunk (Fig. 1B′), and two had double outlet right ventricle (DORV) (Fig. 1B″). All embryos had a VSD, with the exception of one embryo, which had an apparently normal heart (Fig. 1C′; Fig. S1). In addition, we examined histologically a set of five control and three vB12-treated *Tbx1^neo2^*^/−^ embryos at E15.5. All control embryos had VSD and PTA, whereas the vB12-treated embryos had VSD and overriding of the aorta, but no PTA (Table 1; Fig. S2).

**Table 1.**
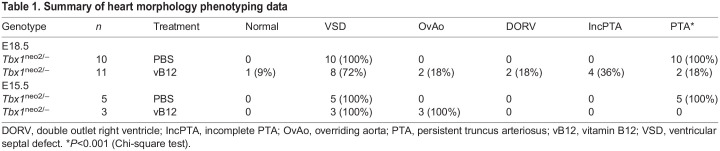
Summary of heart morphology phenotyping data

**Fig. 2.**
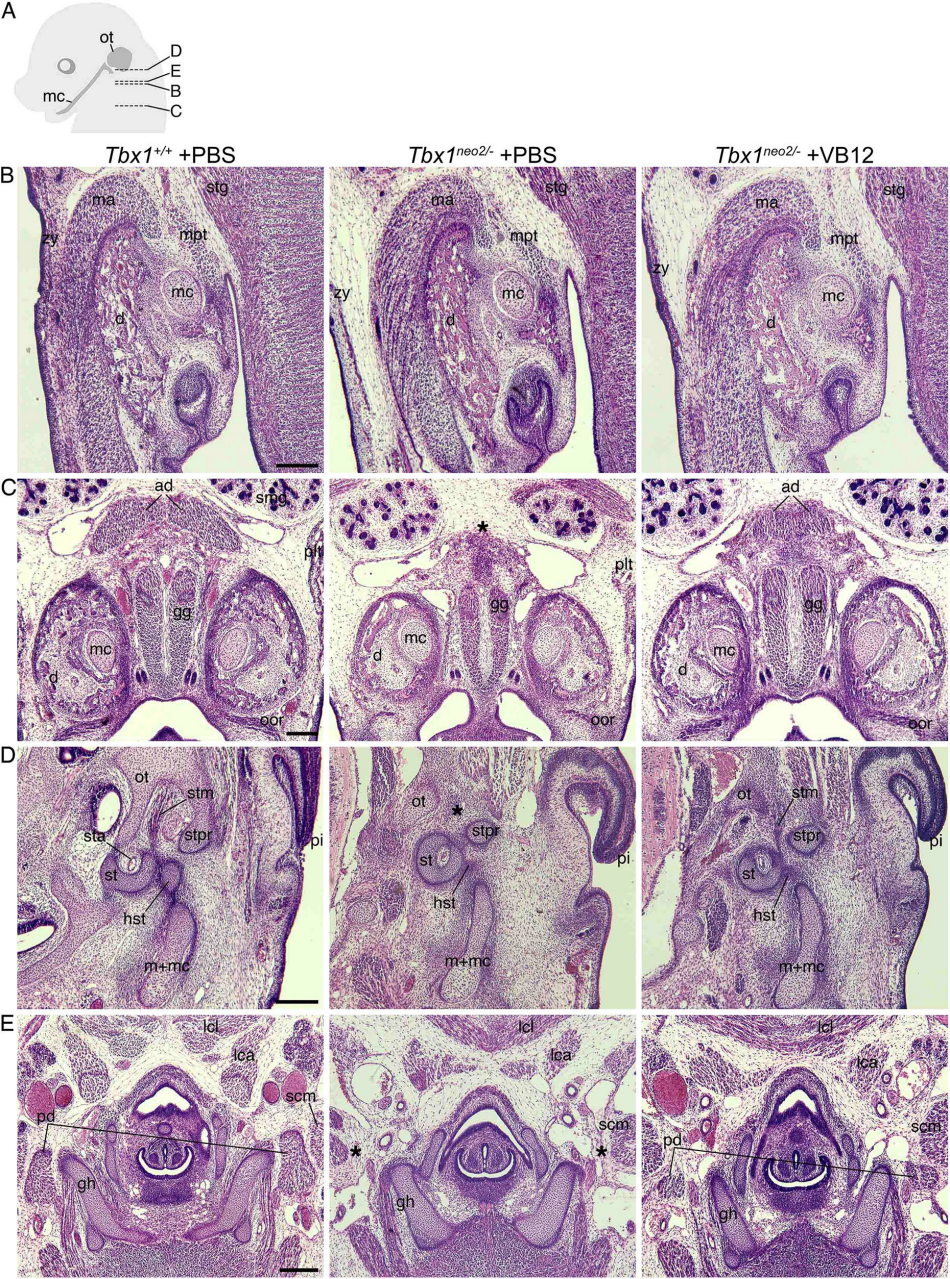
**vB12 treatment partially rescues the branchiomeric muscle phenotype in *Tbx1^neo2/−^* embryos.** Histological sections of *Tbx1*^+/+^ and *Tbx1^neo2^*^/−^ embryos at E15.5, stained with Hematoxylin and Eosin. (A) A diagram showing the section levels. (B,C) Middle (B) and ventral (C) jaw muscles derived from 1st pharyngeal arch (PA). (D,E) Otic (D) and hyoid (E) muscles derived from 2nd PA. The asterisks indicate missing muscles that are rescued by vB12, but not by PBS treatment, in this particular set of embryos (anterior digastric, posterior digastric and stapedius muscles). For a complete list of results, see Table S1. ad, anterior digastric muscle; d, dentary; gg, genioglossus muscle; gh, greater horn of hyoid bone; hst, head of stapes; lca, longs capitis muscle; lcl, longs coli muscle; ma, masseter muscle; mc, Meckel's cartilage; m+mc, malleus and Meckel's cartilage; mpt, medial pterygoid muscle; oor, orbicularis oris muscle; ot, otic capsule; pd, posterior digastric muscle; pi, pinna; plt, platysma muscle; scm, sternocleidomastoid muscle; smg, submandibular gland; st, stapes; sta, stapedial artery; stg, styloglossus muscle; stm, stapedius muscle; stpr, styloid process; zy, zygomaticus muscle. Scale bars: 200 μm.

Reduced dosage of *Tbx1* causes specific craniofacial muscle anomalies (Adachi et al., 2020; Dastjerdi et al., 2007; Kelly et al., 2004). We tested a set of five controls and three vB12-treated *Tbx1^neo2^*^/−^ embryos at E15.5 and scored the craniofacial muscle phenotype (Fig. 2; Fig. S3 and Fig. S4A). Results showed that vB12 treatment reduced the severity of anomalies of the muscles originating from the 1st pharyngeal arch (PA); bilateral defects of the anterior digastric muscles in *Tbx1^neo2^*^/−^ embryos reduced from 60% to 33% after vB12 treatment. Defects of 2nd PA-derived branchiomeric muscles were also rescued by vB12 treatment (Fig. 2; Table S1). Specifically, the number of absent anterior and posterior digastric muscles was significantly lower in the vB12-treated embryos than in PBS-treated controls (*P*>0.05, Fig. S4B). vB12 treatment did not have any effect on muscles derived from more posterior PAs (Figs S3 and S4, Table S1).

### Identification of rescued genes after vB12 treatment *in vivo*

**Fig. 3.**
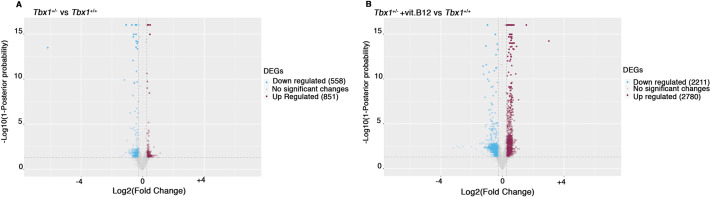
***Tbx1* gene haploinsufficiency alters the expression of 1409 genes, while vB12 treatment in heterozygous background induces dysregulation of 3954 genes.** (A) Volcano plot of significantly upregulated or downregulated genes in *Tbx1^+/−^* whole embryos compared to *Tbx1^+/+^* embryos. Light-blue dots represent downregulated genes; red dots represent upregulated genes. (B) Volcano plot of significantly upregulated or downregulated genes in *Tbx1^+/−^*+vB12 embryos compared to *Tbx1^+/−^*+PBS embryos. Blue dots represent downregulated genes; red dots represents upregulated genes.

**Fig. 4.**
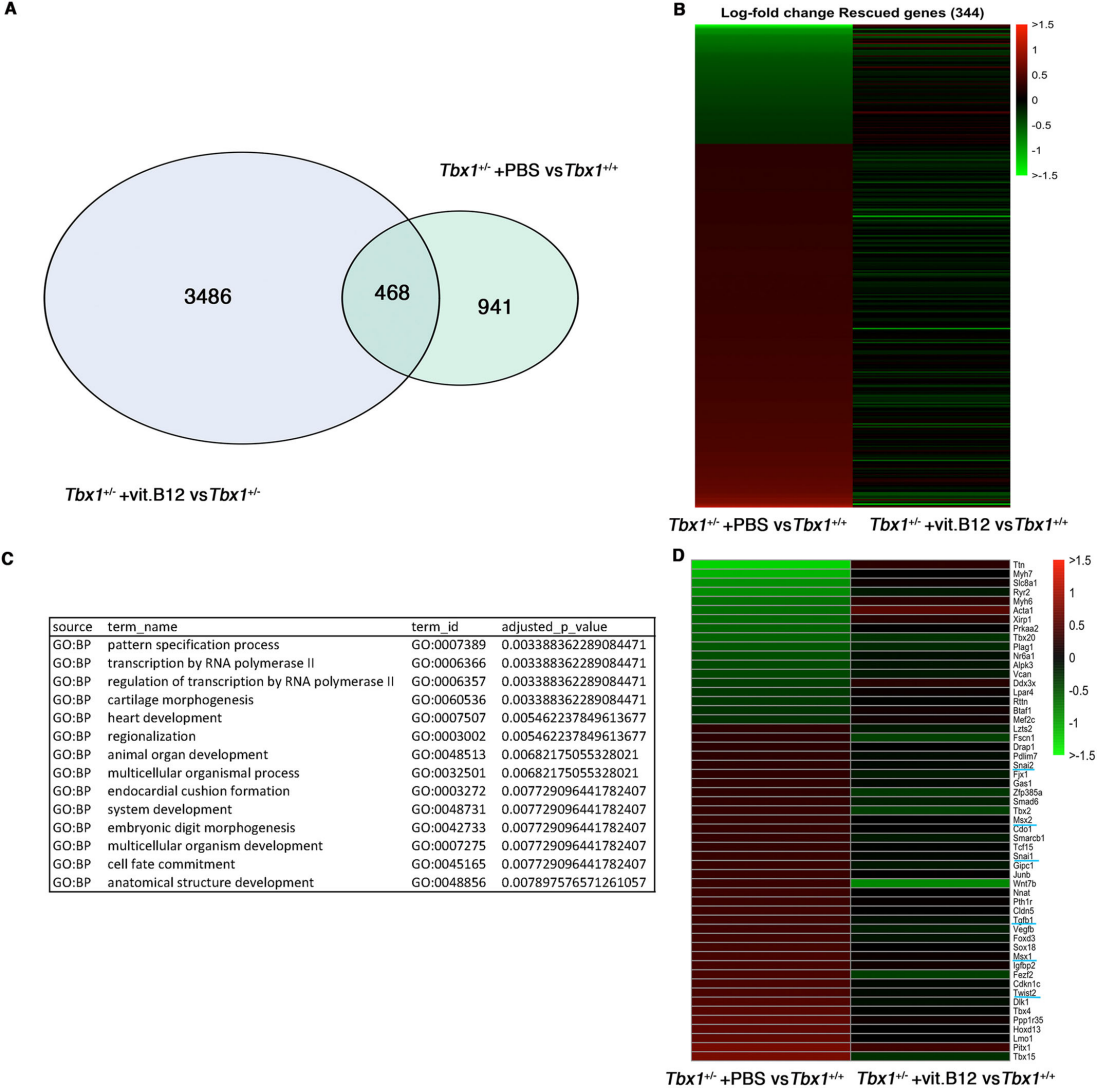
**vB12 rescues the expression of 344 genes involved in gene regulation and heart development.** (A) Venn diagram plot representing the intersection of two groups of differentially expressed genes (*Tbx1*^+/−^ versus *Tbx1*^+/+^, and *Tbx1*^+/−^+vB12 versus *Tbx1*^+/−^+PBS). (B) Heat maps of rescued genes in *Tbx1*^+/−^ embryos (treated with vB12 or PBS) based on their fold change relative to WT. (C) Gene ontology analysis by the gProfiler tool. (D) Heat map of 55 genes known to be expressed in the pharyngeal apparatus, relative to WT.

In order to evaluate the effect of vB12 treatment on embryo tissue transcription, we performed RNA-sequencing (RNA-seq) analysis of whole E9.5 mouse embryos (21-somite stage) after treatment with vB12 or vehicle (PBS) during pregnancy (intraperitoneal injection, 20 mg/kg/day at E7.5, E8.5 and 4 h ahead of the E9.5 harvest). We analyzed the results from PBS-treated wild-type (WT) (*n*=3), PBS-treated *Tbx1*^+/−^ (*n*=3) and vB12-treated *Tbx1*^+/−^ (*n*=2) embryos, where each embryo was sequenced independently and each dataset was treated as a biological replicate. Comparing *Tbx1*^+/−^ and *Tbx1*^+/+^ embryos, we found a total of 1409 differentially expressed genes (DEGs) [fold change cut off>1.2, and posterior probability (PP)>0.95], of which 851 (60.4%) were upregulated and 558 (39.6%) were downregulated (Fig. 3A; Table S2). Gene ontology (GO) analyses of the 851 upregulated genes revealed enrichment of genes involved in oxidative phosphorylation and other metabolic processes, while analyses of the 558 downregulated genes showed enrichment of genes involved in morphogenesis and developmental processes (Table 2). Comparing vB12-treated *Tbx1*^+/−^ embryos with PBS-treated *Tbx1*^+/−^ embryos, we found a total of 3954 DEGs, of which 1862 (47%) were upregulated and 2092 (53%) were downregulated by vB12 treatment (Fig. 3B; Table S2). GO analyses revealed that the upregulated genes were enriched for genes involved in RNA processing, whereas the downregulated genes were enriched for genes involved in developmental processes (Table 3). Details of the GO analyses are provided in Table S3. The intersection of the two groups of DEGs identified 468 shared genes (Fig. 4A), which is a significantly higher number than that expected by chance (*P*=1.4×10^−7^, hypergeometric test). Of these, 344 changed their expression in opposite directions in the two groups, i.e. the mutation changed expression in one direction whereas vB12 treatment rebalanced it (Fig. 4B; genes listed in Table S2); we define these genes as rescued by vB12. In the left column of the heat map shown in Fig. 4B are represented genes dysregulated by the mutation; the right column shows their expression after vB12 treatment (compared to WT). Thus, the dark color in the right column indicates genes that were expressed at or near WT level after treatment. Of these 344 genes, 85 (24.7%, shown in green) were downregulated, and 259 (75.3%, shown in red) were upregulated, relative to WT (Fig. 4B, left column).

**Table 2.**
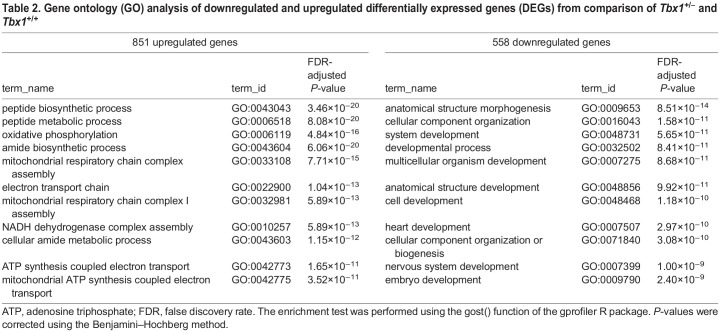
Gene ontology (GO) analysis of downregulated and upregulated differentially expressed genes (DEGs) from comparison of *Tbx1^+/−^* and *Tbx1^+/+^*

**Table 3.**
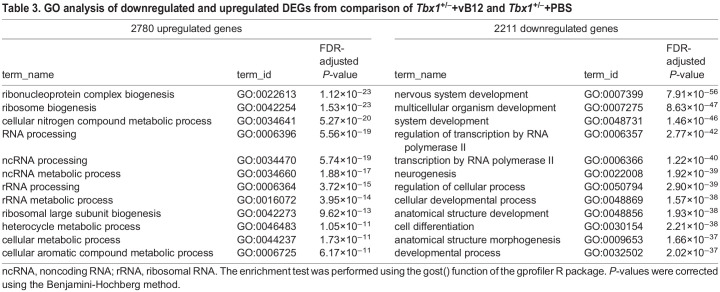
GO analysis of downregulated and upregulated DEGs from comparison of *Tbx1^+/−^*+vB12 and *Tbx1^+/−^*+PBS

We then applied a hypergeometric test to ask whether rescue of gene expression imbalances by vB12 could have occurred by chance, given the high number of genes affected by the treatment. Interestingly, we found that the rescue of the 259 upregulated genes was extremely significant (*P*<<10^−10^), while the rescue of the 85 downregulated genes was borderline with a chance event (*P*=0.052). In addition, the number of genes that were further dysregulated by vB12 (468−344=124), which were almost equally distributed among upregulated and downregulated (59 and 65, respectively) genes, was not significantly different from that expected from a chance event. Thus, the most significant rescue effect of vB12 was on genes that were upregulated by *Tbx1* heterozygosity and downregulated by vB12 treatment. GO of the 344 rescued genes showed enrichment of heart development genes (*P*=0.005) and transcription regulator genes (*P*=0.003) (Fig. 4C). Full results from the GO analysis are provided in Table S3.

### SNAI2 identifies a mesodermal population partially overlapping with but distinct from the TBX1^+^, ISL1^+^ and Mef2c-AHF-Cre^+^ populations

**Fig. 5.**
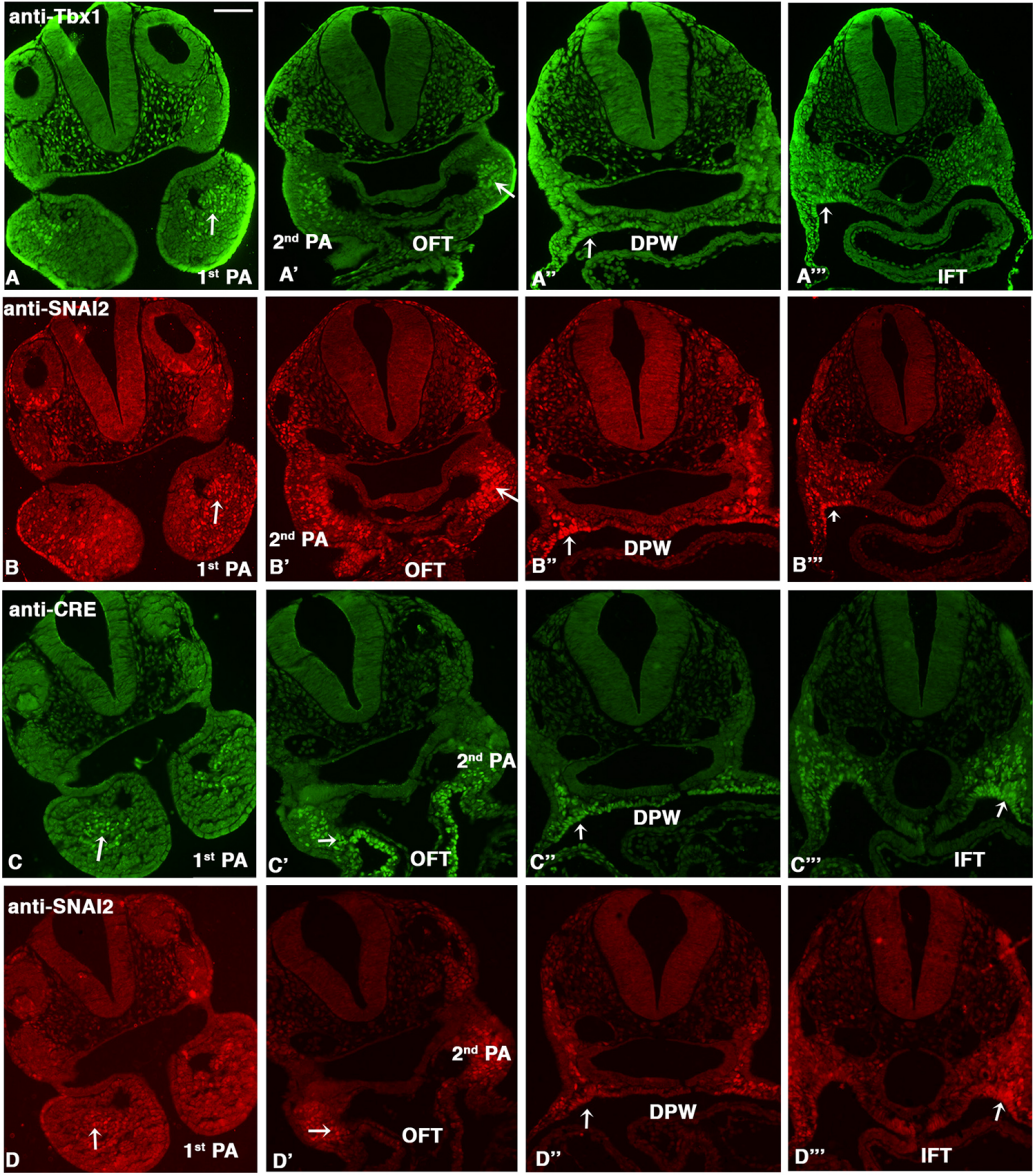
**SNAI2 is expressed in the mesoderm and partially overlaps with TBX1 and Mef2c-AHF expression.** (A-D″) Immunofluorescence (IF) of transverse sections of E9.5 embryos. (A,B) Comparison of TBX1 and SNAI2 expression detected by anti-TBX1 antibody (A) or anti-SNAI2 antibody (B) on the same sections. (C,D) Comparison of the expression patterns of Cre driven by Mef2c-AHF-Cre and SNAI2, detected by anti-Cre antibody (C) and anti-SNAI2 antibody (D). For both comparisons, we used sequential staining because the antibodies were raised in the same species. The arrows indicate the expression of TBX1 or CRE or SNAI2 in core mesoderm region in the 1st PA, in the distal mesoderm in the 2nd PA, in the dorsal pericardial wall and in the second heart field. DPW, dorsal pericardial wall; IFT, inflow tract; OFT: outflow tract; PA, pharyngeal arch. Scale bar: 100 µm.

Among the rescued genes, we identified 55 genes known to be expressed in the CPM, or its derivatives, and to a lesser extent in other tissues of the PhAp (Fig. 4D; Table S2). Among these, we noted a set of genes known to be involved in the Tgfβ1 pathway. Specifically, *Snai1*, *Snai2*, *Twist2*, *Msx1* and *Tgfb1* were all upregulated in *Tbx1*^+/−^ mutant embryos and downregulated by vB12 treatment (blue underlined in Fig. 4D). We selected *Snai2*, encoding a transcriptional repressor also known as SLUG, which has not been previously associated with TBX1 biology. We performed immunofluoresence (IF) with an anti-SNAI2 antibody (Hultgren et al., 2020) to determine its expression relative to markers of the CPM in E9.5 WT embryos. The anti-SNAI2 and anti-TBX1 antibodies are raised in the same species; therefore, we used them in sequence on the same sections. Similar results were obtained by inverting the order of the antibodies. As shown in Fig. 5A,B, at all section levels considered, there was a very similar distribution of the two proteins, with notable exceptions. Specifically, in the 1st PA, both proteins were present in the core mesoderm, but SNAI2^+^ cells were fewer in the core, and there were more of them scattered in the body of the PA (arrow in Fig. 5B); TBX1^+^ cells were more evident in the proximal region of the arch (Fig. 5A,B). In the 2nd PA, the SNAI2 domain extended more distally towards the OFT, compared to the TBX1 domain (Fig. 5A′,B′). At the other two levels analyzed, namely, posterior to the OFT (Fig. 5A″,B″) and immediately anterior to the inflow tract (IFT) (Fig. 5A‴,B‴), the distribution of the two proteins was very similar in the lateral aspects of the dorsal pericardial wall and splanchnic mesoderm.

Next, we compared the expression of SNAI2 to that of the Mef2c-AHF enhancer using an anti-CRE antibody (also raised in the same species as the anti-SNAI2 antibody) on sections of Mef2c-AHF-Cre embryos (Verzi et al., 2005). Also in this case, the expression patterns of the two proteins were very similar (Fig. 5C,D), except for two substantial differences: in the 2nd PA, Mef2c-AHF-Cre was expressed more distally towards the OFT, including the myocardial layer of the OFT, (Fig. 5C′,D′), more extensively in the dorsal pericardial wall (Fig. 5C″) and more extensively in the splanchnic mesoderm of the posterior region of the embryonic pharynx (Fig. 5C‴,D‴).

**Fig. 6.**
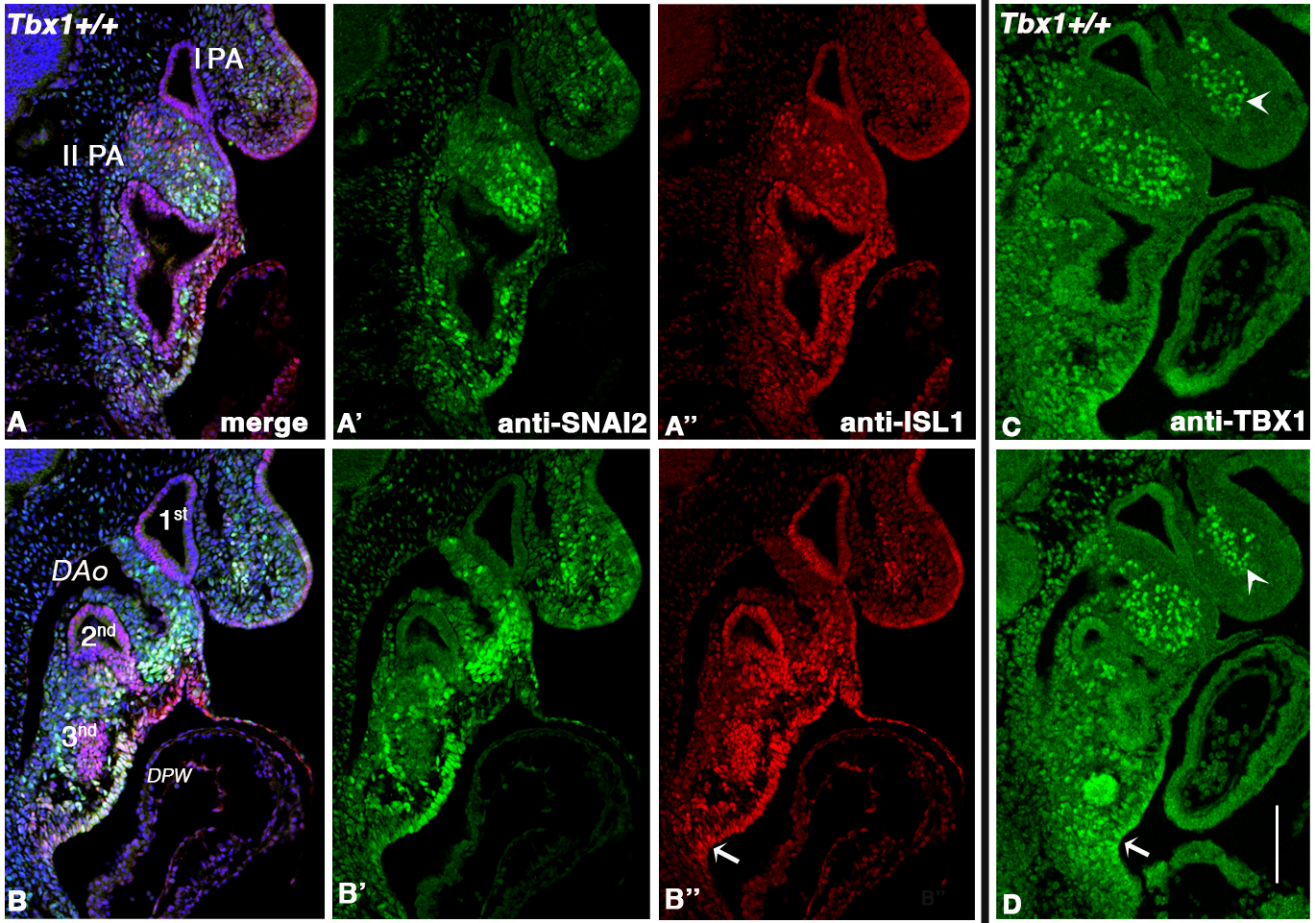
**SNAI2 and ISL1 have a similar pattern of expression with exceptions in the proximal 2nd PA and posterior second heart field.** (A-B″) Double IF with anti-SNAI2 and anti-ISL1 on sagittal sections of WT E9.5 embryos. Two representative images from lateral (A) to medial (B), with immunostaining by anti-SNAI2 antibody (green) and anti-ISL1 antibody (red). Arrows indicate regions in which ISL1 expression is more extensive than SNAI2 expression (compare A′ with A″, and B′ and B″. (C,D) Similar sections to those in A and B, respectively, immunostained with an anti-TBX1 antibody. Note similar expression of TBX1 and ISL1 at this level (arrows). Note also the difference in the expression of TBX1 in the 1st PA (arrowheads) compared to both ISL1 and SNAI2. DAo, dorsal aorta; DPW, dorsal pericardial wall; I PA, 1st pharyngeal arch; II PA, 2nd pharyngeal arch. Scale bar: 200 µm. Fig. S5 shows a double-stained, higher-magnification image of a section adjacent to the section shown in A.

**Fig. 7.**
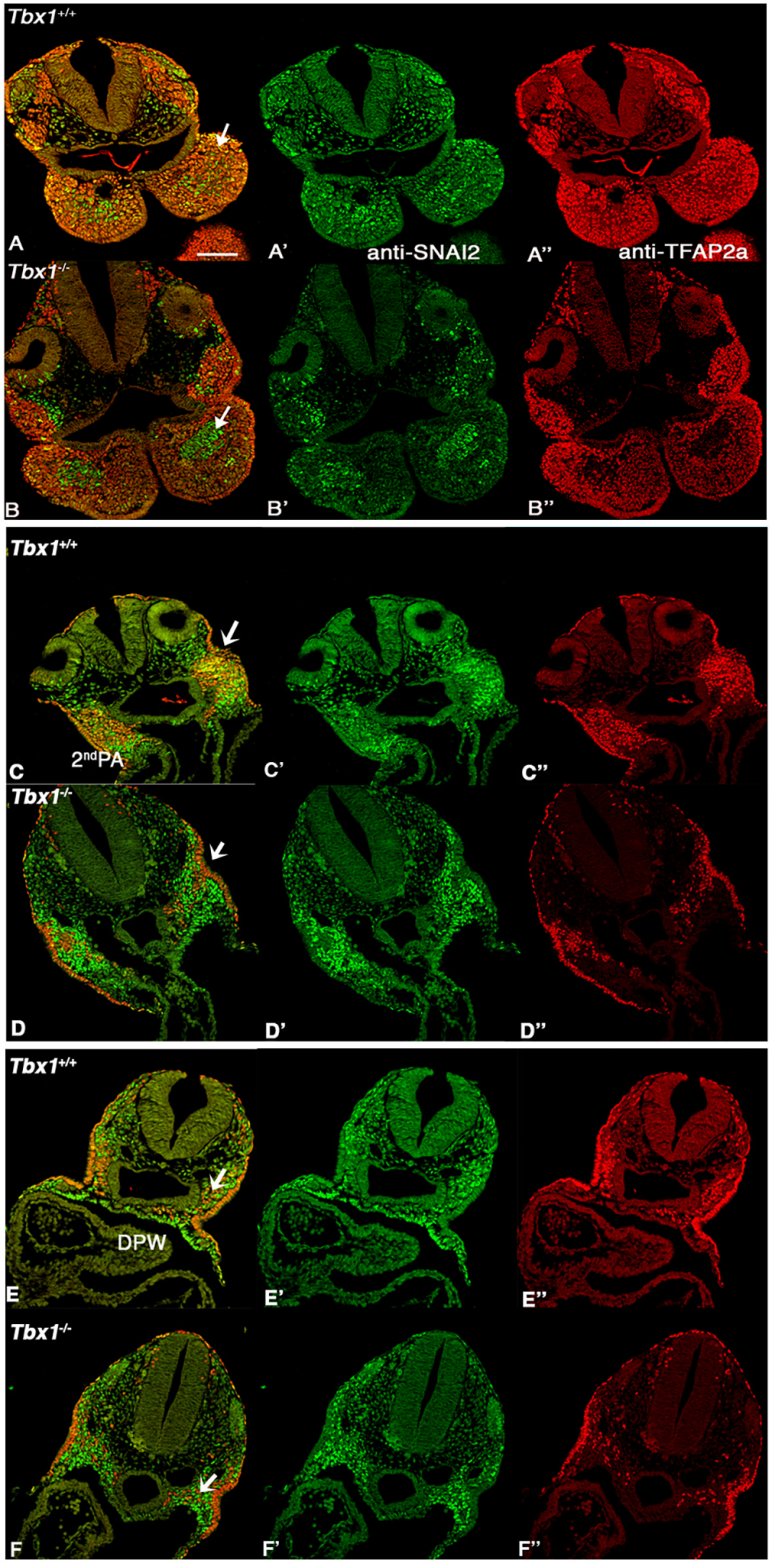
**TFAP2A and SNAI2 highlight regionalization defects in *Tbx1^−/−^* embryos.** (A-F″) Representative images of double IF with immunostaining by anti-TFAP2 (red) and anti-SNAI2 (green) antibodies on transverse sections of *Tbx1*^+/+^ (A,C,E) and *Tbx1*^−/−^ (B,D,F) E9.5 embryos at the level of the 1st PA (A,B), 2nd PA/OFT (C,D), and DWP between the OFT and IFT (E,F). In general, there is minimal or no overlap between the two markers. In A and B, note the different relative distribution of SNAI2^+^ and TFAP2A^+^ cells in the 1st PA (arrows). In C and D, cells of the two lineages are intermingled in the WT 2nd PA but segregated in the mutant (arrows); in E and F, note the expansion of SNAI2 expression in the mutant (arrows). Scale bar: 200 µm.

We then compared SNAI2 expression to that of ISL1, which is expressed throughout the CPM (Fig. 6). The two markers had a very similar, mostly overlapping expression in the 1st PA (Fig. 6A,B) of WT embryos. In the 2nd arch, SNAI2 was more highly expressed in the distal portion of the arch, where it overlapped with ISL1, whereas in the proximal portion, there was a high expression of ISL1 but not of SNAI2. Double-labeled cells were detected in a mediolateral region of the dorsal pericardial wall, where ISL1 expression was more extensive (Fig. 6A″,B″); in addition, the more posterior expression of ISL1 in the splanchnic mesoderm (arrow in Fig. 6B″) is similar to the TBX1 expression domain (Fig. 6C,D). Fig. S5 shows a higher-magnification image of a section adjacent to that shown in Fig. 6A. Because of the scattered expression of SNAI2 in the body of the 1st PA, which is heavily populated by NCCs, we co-stained E9.5 embryos (WT) with TFAP2A, which labels migrating NCCs at this stage (as well as ectodermal cells) (Mitchell et al., 1991). With very few exceptions, we did not observe double-labeled cells in the entire embryo (examples in Fig. 7A,C,E), but we observed extensive intermingling of SNAI2^+^ and TFAP2A^+^ cells. Thus, SNAI2 is expressed in mesodermal cells, mainly in the distal 2nd PA, in the mediolateral dorsal pericardial wall, in the posterior lateral splanchnic mesoderm and, to a minor extent, in the 1st PA.

### *Tbx1* mutants have lineage regionalization abnormalities

We next investigated whether *Tbx1* loss of function affected the SNAI2^+^ population. We first examined this population in comparison to NCCs (TFAP2^+^; TBX1^−^). At the level of the 1st PA, we found a striking pattern in which SNAI2^+^ cells in *Tbx1*^−/−^ embryos were tightly grouped in the core mesoderm, forming a large area surrounded by, but not mixed with, TFAP2A^+^ cells (Fig. 7B), whereas in WT embryos the two cell types were intermingled (Fig. 7A). A similar pattern was evident in the head mesoderm/proximal 1st PA (Fig. 7A,B, arrows). At the level of the 2nd PA, which is severely hypoplastic in *Tbx1*^−/−^ embryos, the mixing of the two populations was substantially reduced, although here the TFAP2^+^ population appeared smaller than in WT (Fig. 7C,D, arrows). More posteriorly (caudal to the OFT), this segregation phenotype was not apparent; of note is a relative expansion of the SNAI2^+^ population at this level in the splanchnic mesoderm of the mutant embryo (Fig. 7E,F, arrows).

**Fig. 8.**
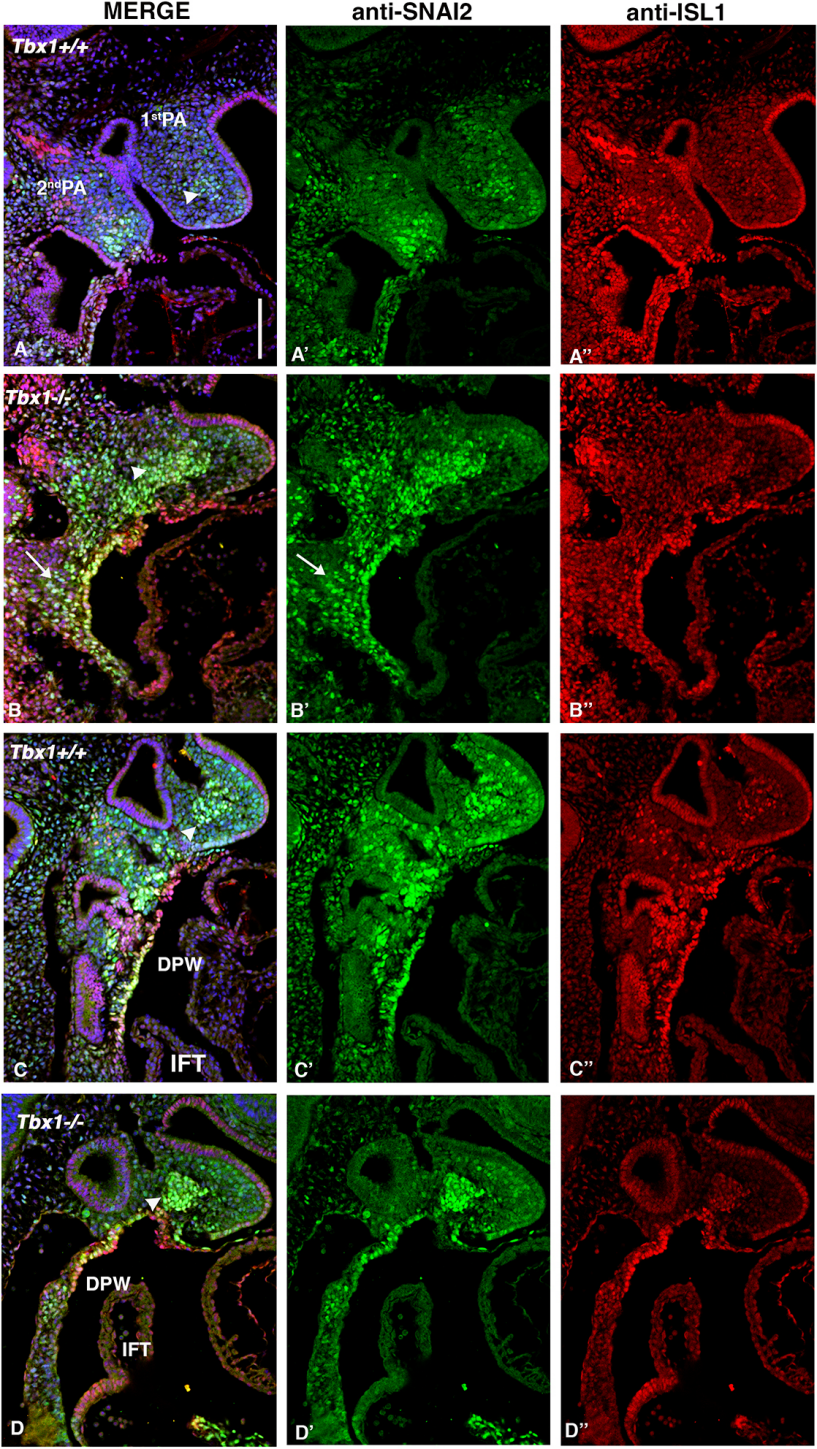
**The core mesoderm of the 1st PA of *Tbx1^−/−^* embryos is populated by SNAI2^+^;ISL1^+^ cells.** (A-D″) Double IF using anti-SNAI2 (green) and anti-ISL1 (red) antibodies on lateral (A,B) and mediolateral sagittal (C,D) sections of the 1st PA from *Tbx1*^+/+^ (A,C) and *Tbx1*^−/−^ (B,D) embryos. Note the colocalization of ISL1 and SNAI2 in the core mesoderm of the 1st PA in *Tbx1*^−/−^. In the WT, this is much less evident (compare regions indicated by arrowheads in A, B, C and D). In addition, SNAI2 expression is extended in the splanchnic mesoderm of the *Tbx1*^−/−^ embryo (arrows in B and B′). Scale bar: 100 µm.

We next examined the distribution of SNAI2^+^ cells compared to ISL1^+^ cells in *Tbx1*^−/−^ embryos. In the 1st PA of *Tbx1*^−/−^ embryos, and in contrast to WT embryos, we observed a large, well-defined cluster of SNAI2^+^ cells that were mostly ISL1^+^ in the core mesoderm and appeared to extend posteriorly, as if it resulted from merging with the core of the 2nd PA, which is severely hypoplastic in these mutants (arrowheads in Fig. 8A,B). In a more medial sagittal plane, the aggregate is also clearly visible (Fig. 8C,D). This aggregation phenotype was observed in all the homozygous embryos examined (*n*=11).

**Fig. 9.**
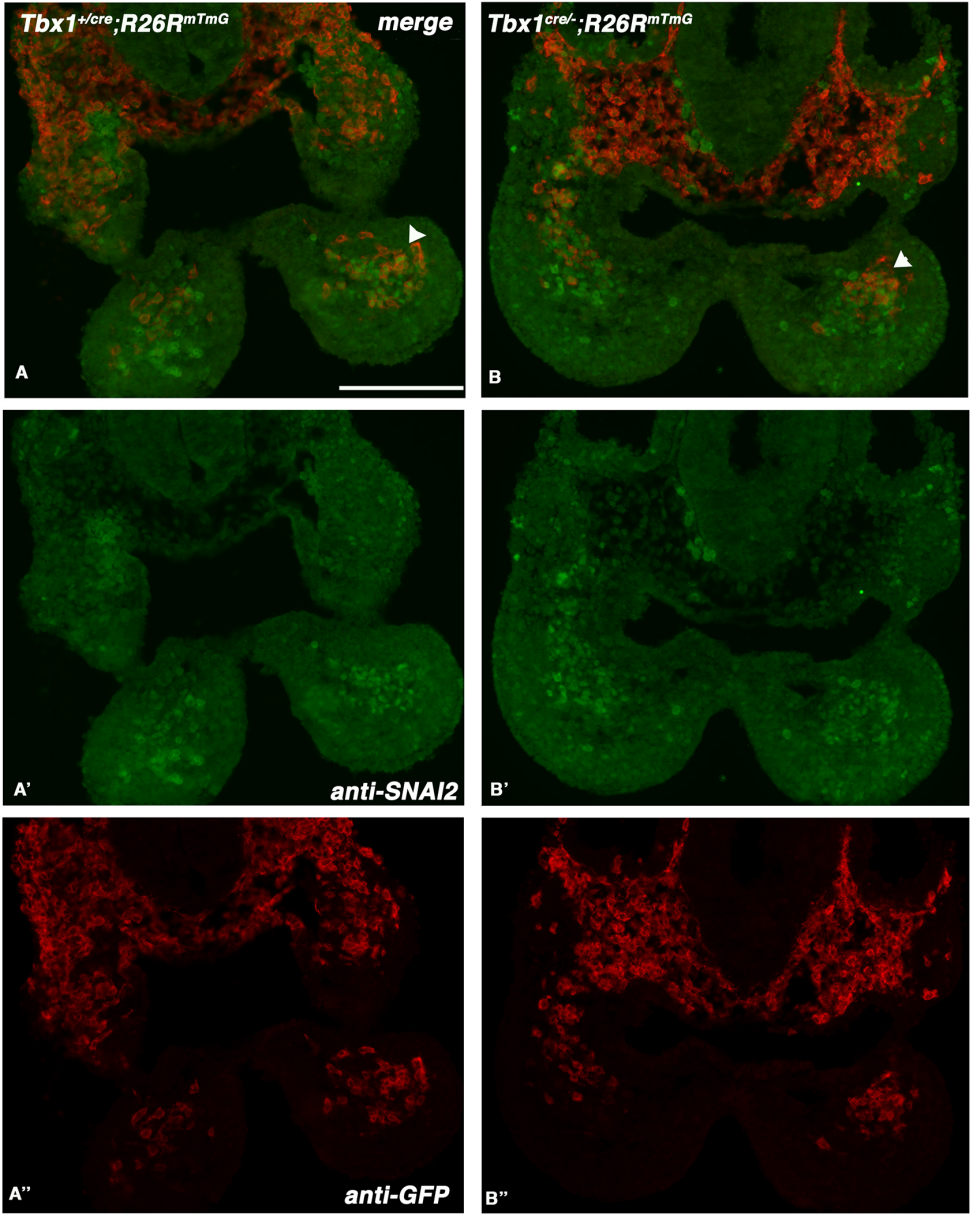
**The positional relationship between TBX1^+^ and SNAI2^+^ cells is maintained in *Tbx1* null embryos.** (A-B″) IF using anti-GFP (red) and anti-SNAI2 (green) antibodies on transverse sections of *Tbx1^Cre^*^/+^;R26R^mTmG^ (functionally heterozygous; A) and *Tbx1^Cre^*^/−^;R26R^mTmG^ (null mutant; B) E9.5 embryos. A′,B′ and A″,B″ show the green and red, respectively, signals in separate channels. Note that *Tbx1*-activating cells and their descendants (in red) localize predominantly laterally in the core mesoderm (arrowheads) and in the proximal region of the PA, in both cases. Scale bar: 100 µm.

We next tested whether cells of the *Tbx1* genetic lineage are mislocalized, relative to SNAI2^+^ cells, in the absence of *Tbx1* function. To this end, we performed anti-SNAI2 and anti-GFP IF on *Tbx1^Cre^*^/−^;*R26R^mT-mG^* (*Tbx1* null) and *Tbx1^Cre^*^/+^;*R26R^mT-mG^* (heterozygous, control) E9.5 embryos (*Tbx1^Cre^* is a null allele). Results showed that, in control embryos, GFP^+^ cells (shown in red in Fig. 9A,B,A″,B″) were more prominent in the proximal and lateral aspects of the 1st PA relative to SNAI2^+^ cells, with only a limited overlap. Moreover, the relationship between the markers was largely conserved in *Tbx1* null embryos (Fig. 9B), indicating that the SNAI2^+^ aggregate in the 1st PA is mostly made of cells that did not activate *Tbx1* gene transcription.

**Fig. 10.**
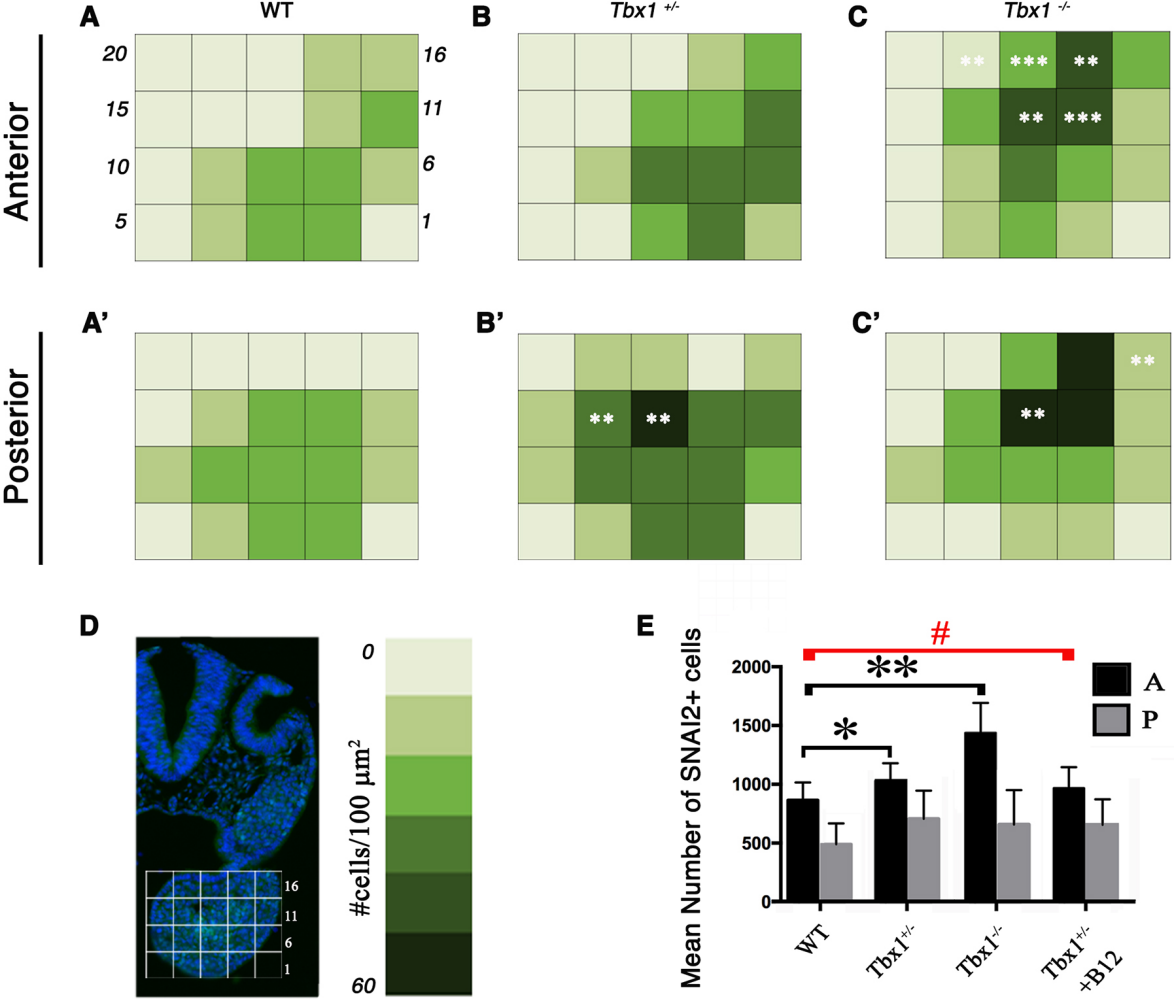
**Summary of quantitative analyses of SNAI2 signal on the 1st PA of the genotypes indicated.** (A,B,C) Color-coded visualization of SNAI2^+^ cell density in 20 subregions of the 1st PA, anterior segment: the darker the color the higher the density, as illustrated in the color map of D. (A′,B′,C′) Color-coded distribution in the posterior segment. (D) Scheme of the 5×4 grid used for cell counting (left), and color-coded map of density measurements (right). (E) The histogram shows total cell counts of SNAI2^+^ cells in the anterior (black) and posterior (gray) segments. *P<0.05; **P<0.01; ***P<0.001; #, no statistical significance (unpaired Student's *t*-test, one-tailed).

To support the qualitative observations reported above, we performed quantitative evaluation of SNAI2^+^ cell number and distribution in WT, *Tbx1*^+/−^ and *Tbx1*^−/−^ mutant embryos. For these tests, we used an additional set of five embryos/genotype after SNAI2 IF. Consecutive images of transverse sections were collected along the entire 1st PA. Using computer-generated 5×4 grids placed on the arch images, we divided each section into 20 subregions, and we counted the SNAI2^+^ cells for each subregion and for each 10 µm-thick section. Subsequently, we evaluated the numerosity (absolute number of positive cells) and density (number of cells per 100 µm^2^) for each region and genotype. Because of differences in the spatial distribution of SNAI2^+^ cells in the WT, we divided the arch into two segments along the anterior–posterior axis. The anterior (A) segment is defined from the early invagination of the first branchial pouch (upper limit of the arch) through the buccopharyngeal membrane (lower limit); the rest of the arch is the posterior (P) segment. A graphic representation of the results is shown in Fig. 10. In the A segment, the cell density distribution is significantly affected by *Tbx1* dosage (WT versus *Tbx1*^+/−^, *P*<0.001; WT versus *Tbx1*^−/−^, *P*<0.001). Furthermore, we observed subregions of high SNAI2^+^ cell density in the *Tbx1*^−/−^ embryos that were not observed in other genotypes (Bin 12, *P*=0.0003; Bin 13, *P*=0.00345: Bin 17, *P*=0.005; Bin 18, *P*=0.0005; Bin 19, *P*=0.007). In addition, the numerosity of cells was significantly different across genotypes (WT versus *Tbx1*^+/−^, *P*=0.048; WT versus *Tbx1*^−/−^, *P*=0.004). In the P segment, the SNAI2^+^ cell density increased significantly with the reduction of *Tbx1* dosage (WT versus *Tbx1*^+/−^, *P*<0.001; WT versus *Tbx1*^−/−^, *P*<0.001) (Fig. 10A,B,C). Unlike in the A segment, in the P segment, we found subregions in which cell densities were significantly higher in *Tbx1* heterozygous and null embryos (in *Tbx1*^+/−^: Bin 13, *P*=0.0047; Bin 14, *P*=0.003; in *Tbx1*^−/−^: Bin 12, *P*=0.002; Bin 16, *P*=0.0012) (Fig. 10A′,B′,C′). Furthermore, cell counts revealed that, overall, the A segment of both mutants had a higher number of SNAI2^+^ cells compared to WT, while there were no significant differences in the number of cells in the P segment (Fig. 10E).

Thus, quantitative data revealed regional differences along the anterior–posterior axis of the 1st PA in the response to *Tbx1* dosage and confirmed the visual evidence that the SNAI2^+^ cell population tends to be denser as the *Tbx1* gene dosage decreases.

To understand how the aggregation of SNAI2^+^ cells in the 1st PA of *Tbx1*^−/−^ mutants arises, we examined earlier developmental stages: 11-, 15- and 20-somite stage (st), immunostained with anti-SNAI2 and anti-ISL1 antibodies. In the WT embryo, at 11st, the 1st PA was mostly populated by compacted mesoderm (ISL1^+^ and SNAI2^+^) and by a very limited non-mesodermal mesenchymal population (Fig. S6A,A′). At this stage, the NCCs have not populated the arch in a substantial manner (Grenier et al., 2009). As NCCs populate the arch at 15st, they mostly surround the mesodermal core, but a subpopulation invades the core, resulting in the dispersal of SNAI2^+^ and ISL1^+^ cells within the arch mesenchyme (Fig. S6B,B′). This process of dispersion continues at 20st (Fig. S6C,C′). In the *Tbx1*^−/−^ embryo, this process of dispersion does not occur at any stage (Fig. S6D-F′), and, as a result, the SNAI2^+^ and ISL1^+^ cells remain compacted. These observations suggest that, in *Tbx1* mutants, NCCs fail to penetrate the pre-existing mesodermal core so that the two lineages remain segregated.

**Fig. 11.**
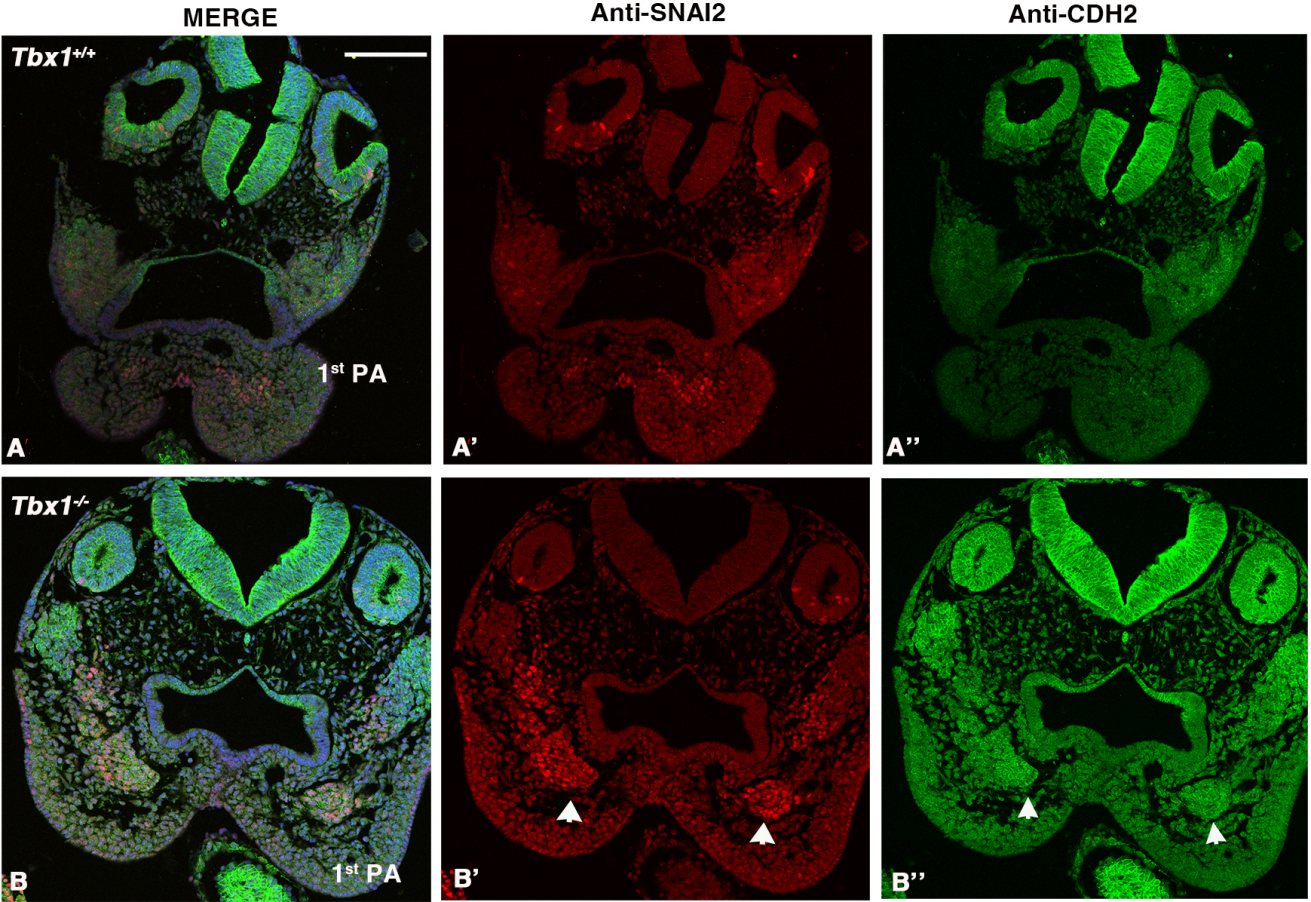
**CDH2 (N-cadherin) is upregulated in the 1st PA of *Tbx1^−/−^* E9.5 embryos.** (A-B″) IF of transverse sections of E9.5 embryos with the genotype indicated, double labeled with anti-SNAI2 (red) and anti-CDH2 (green) antibodies. (A-A″) Transverse sections of a WT embryo at the level of the 1st PA. (B-B″) Transverse sections of a *Tbx1*^−/−^ embryo at the level of the 1st PA; note the upregulation of CDH2 on the SNAI2^+^ aggregate at the core of the arch (arrowheads). Scale bar: 25 µm.

We next asked whether the segregation of SNAI2^+^ cells may be explained by differential cell–cell adhesion mediated by cadherins. CDH2 (also known as N-cadherin) is expressed in many mesodermal tissues and is involved in collective cell migration (reviewed in Alimperti and Andreadis, 2015). We performed IF with an anti-CDH2 antibody along with an anti-SNAI2 antibody, and we found very low expression in the 1st PA of WT E9.5 embryos (Fig. 11A). However, in *Tbx1*^−/−^ embryos, the compacted SNAI2^+^ mesodermal core of the 1st PA was clearly CDH2^+^, well above the level of expression in the surrounding mesenchyme (Fig. 11B).

In summary, our expression analysis indicates that TBX1 has cell-autonomous, and perhaps more extensive non-cell-autonomous, functions in regulating the regionalization of cell lineages that are critical for the development of the PhAp.

### SNAI2 identifies a novel haploinsufficiency phenotype rescued by vB12

**Fig. 12.**
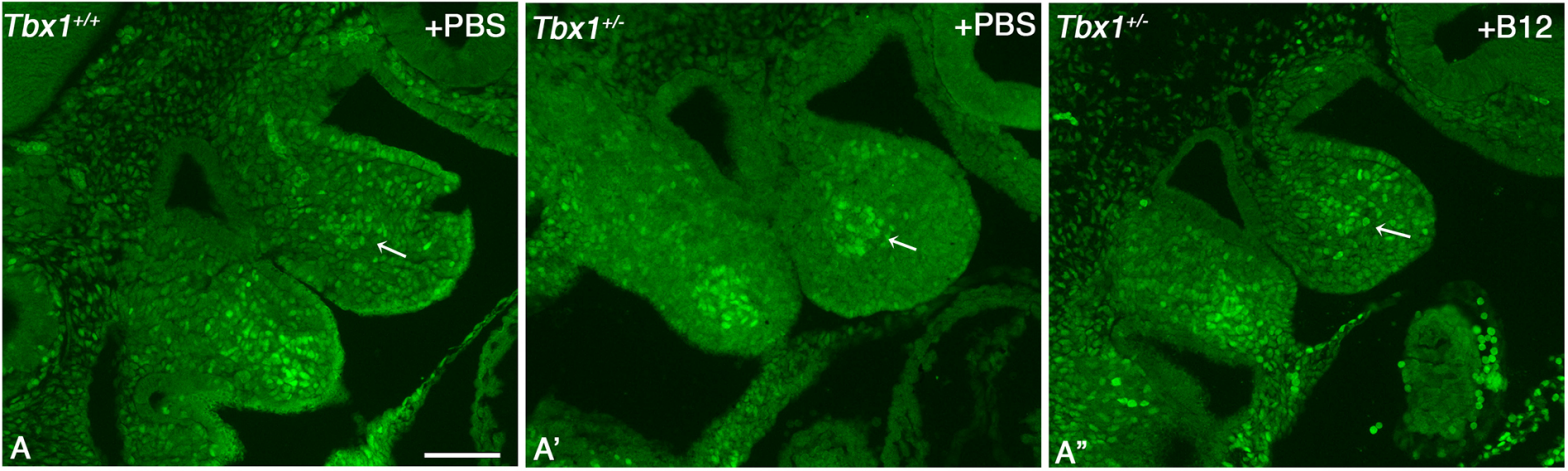
***Tbx1* gene haploinsufficiency causes SNAI2^+^ cell condensation in the 1st PA rescued by vB12.** (A-A″) Images of IF with an anti-SNAI2 antibody on WT+PBS (A), *Tbx1*^+/−^+PBS (A′) and *Tbx1*^+/−^+vB12 (A″) E9.5 embryos. Mediolateral sagittal sections. A cluster of SNAI2^+^ condensed cells in the core mesoderm is noticeable in A′; in A″, cells are more disperse, more similar to the WT pattern. Additional examples of the condensation phenotype are shown in Fig. S6. Scale bar: 100 µm.

*Tbx1*^−/−^ embryos exhibit significant anatomical anomalies, thus raising the question of whether some of the regionalization differences may be due to anatomical constraints. However, quantitative data shown in Fig. 10 indicate that heterozygous embryos, which have no gross anatomical abnormalities (with the exception of hypoplasia of the 4th PA artery and parathyroids), also have regionalization anomalies. Furthermore, visual IF analysis showed that, in *Tbx1*^+/−^ embryos, SNAI2^+^ cells were grouped in the core mesoderm (Fig. 12A′; additional examples shown in Fig. S7). A similar result was obtained using *Mef2c-AHF-Cre*-driven deletion of *Tbx1* in *Tbx1^flox^*^/+^;*Mef2c-AHF-Cre* embryos (Fig. S8), indicating that this anomaly is dependent upon *Tbx1* haploinsufficiency in the mesoderm. This phenotype is reminiscent of, but less severe than, that noted in *Tbx1*^−/−^ embryos (compare with Fig. 7A,B and Fig. 8C,D). Interestingly, vB12 treatment re-established a staining pattern similar to WT (Fig. 12A″) in three independent experiments. Quantitative evaluation of the SNAI2 staining on an additional set of four embryos revealed that the number of SNAI2^+^ cells increased modestly, but significantly, in the A segment of PBS-treated heterozygous mutants, compared to PBS-treated WT embryos (Fig. 10E). vB12 treatment improved the numerosity phenotype, as it became closer to the WT phenotype (because there is no significant difference with the WT embryos), but not sufficiently to reach significance when tested against the untreated heterozygous embryos (*P*=0.5, Mann–Whitney one-tailed test). We think that this is because our quantitative test is not sufficiently sensitive. Therefore, the phenotypic improvement after vB12 treatment is documented in part by qualitative observations and in part by quantitative evidence that treated embryos are closer to the WT phenotype than the untreated ones.

## DISCUSSION

Gene haploinsufficiency is a frequent cause of birth defects and postnatal morbidity. Counterbalancing haploinsufficiency is possible, but it is challenging in the clinical setting and may lack sufficient precision to rescue the full spectrum of phenotypic changes. Pharmacological rescue would be particularly useful in the clinics if it could be precisely targeted.

High doses of vB12 can partially rescue the 4th PA artery phenotype associated with *Tbx1* gene haploinsufficiency in the mouse (Lania et al., 2016); in this study, we tested additional phenotypic recoveries by crossing a hypomorphic allele, which, combined with a null allele, causes a more complex phenotype than the one exhibited by heterozygous mutants. Indeed, we observed that vB12 treatment improved the septation process of the outflow tract in *Tbx1^neo2^*^/−^ embryos. In addition, we found that branchiomeric muscle defects were also diminished after treatment.

Having shown that the treatment improves a range of phenotypic abnormalities, we sought to leverage this property to identify genes and pathways dysregulated by *Tbx1* haploinsufficiency and rebalanced by vB12; these genes may be critical for the pathogenesis of the rescued phenotypes. We found that, in E9.5 *Tbx1*^+/−^ embryos, 24% of the genes dysregulated by *Tbx1* haploinsufficiency were rescued by vB12, and GO analysis of the rescued genes revealed enrichment of genes involved in heart development, thus providing a transcriptional correlate of the phenotypic observations. Among the rescued genes, we noted several that are implicated in epithelial–mesenchymal transition, and we selected *Snai2* for further studies. At E9.5, SNAI2 identifies a mesodermal population in the PhAp that partially overlaps with other markers of the CPM, such as TBX1, ISL1 and Mef2c-AHF-Cre. This suggests that the SNAI2^+^ cell population may include SHF cardiac progenitors and branchiomeric muscle progenitors of the cardiopharyngeal lineage. Importantly, the SNAI2 expression pattern changes in heterozygous and homozygous *Tbx1* mutants. This could be due to ectopic expression of the *Snai2* gene, or to mislocalization of SNAI2^+^ cells. However, the second hypothesis, i.e. defective regionalization, is supported by the finding that the expression of other genes also follows similar pattern changes. In addition, quantitative analyses of SNAI2^+^ cells confirmed significant distribution and density changes, and revealed an absolute increase in the number of SNAI2^+^ cells in the 1st PA of *Tbx1*^−/−^ embryos at E9.5. In addition, the finding that NCCs, as identified by TFAP2A staining, are also mislocalized supports the hypothesis that the *Tbx1* mutation is associated with anomalous regionalization of multiple cell lineages. It is unlikely that regionalization anomalies are due to morphogenetic defects because some of these anomalies, along with gene expression dysregulation, are also evident in the heterozygous mutants that do not show major morphogenetic defects.

The aggregation of SNAI2^+^ cells, particularly evident in the 1st PA, and the segregation of these cells from the neural crest lineage, suggest that the mutation is altering mechanisms of cell sorting, a crucial process in embryonic morphogenesis. This problem may occur for a number of reasons. For example, the differential adhesion hypothesis (Steinberg, 1962) predicts that cells tend to group together if they have higher affinity with each other, compared with other neighboring cell populations. This possibility is supported by the finding that aggregated cells express higher levels of CDH2, a cell adhesion molecule, compared to the surrounding NCC-derived mesenchyme in the 1st PA of *Tbx1*^−/−^ embryos. Our observations in the 1st PA of early embryos indicate that, in *Tbx1* mutants, incoming mesenchymal cells fail to mix with core mesodermal cells, consistently with a differential cell adhesion hypothesis. Intermingling of Myf5^+^ myogenic core cells and incoming TFAP2A^+^ NCCs in the 1st PA of WT embryos has previously been described (Grenier et al., 2009), although the mechanisms that govern this process are not yet established. We show here that TBX1 function is part of these mechanisms, although the effectors remain to be identified. The association of *Tbx1* heterozygosity and NCC distribution has been noted previously in the posterior pharynx (Calmont et al., 2009). Cell–cell adhesion and/or cell–extracellular matrix interactions may interfere with NCC migration, delaying proper localization, and TBX1 loss of function has been associated with alteration of these interactions (Alfano et al., 2019). Treatment with vB12 could also target NCCs and modify their migratory behavior.

It is tempting to speculate that lineage regionalization abnormalities are part of the pathogenetic mechanism underlying the severe developmental defects of the PhAp associated with *Tbx1* mutation. Mislocalization, even transient, of different cell types may expose them to different signaling cues (or different concentrations thereof), causing further developmental defects downstream.

In this work, we used *Snai2* as a marker gene, but we did not address a potential role of *Snai2* in the observed phenotypes. SNAI2 is a transcriptional repressor that targets genes encoding adhesion molecules (such as E-cadherin) in epithelial cells, thus supporting their mobilization and mesenchymalization (Zhou et al., 2019). It is difficult to directly apply these concepts to the pharyngeal mesenchymal cells that we have studied. However, SNAI2 has also been associated with a number of different functions, including skeletal muscle differentiation (Tang et al., 2016) and with upregulated CDH2 in some contexts (reviewed in Loh et al., 2019). Therefore, it would be of interest to determine, in the future, whether SNAI2 has a specific role in branchiomeric muscle differentiation or development, which is impaired in *Tbx1* mutants (Grifone et al., 2008; Kelly et al., 2004). However, at this point we do not have evidence implicating *Snai2* upregulation in the pathogenesis of the *Tbx1* mutant phenotype.

In summary, we show that vB12 treatment is sufficient to rescue, in part, several anomalies of the PhAp. We leveraged this activity to identify a set of genes already known to be involved in heart development that may be part of or associated with the pathogenesis of TBX1-dependent phenotypes. Finally, one of the rescued genes, encoding SNAI2, has been instrumental in the discovery of a novel phenotype of lineage regionalization defects.

## MATERIALS AND METHODS

### Mouse lines

In this work, we used mouse lines previously described: *Tbx1^lacZ^* (referred to as *Tbx1*^+/−^) (Lindsay et al., 2001), *Tbx1^neo2^* (Zhang et al., 2006), *Tbx1^Cre^* (Huynh et al., 2007), *Tbx1^flox^* (Xu et al., 2004), R26R^mTmG^ (Muzumdar et al., 2007) and Mef2c-AHF-Cre (Verzi et al., 2005). We crossed *Tbx1^lacZ^*^/+^ mice with *Tbx1^lacZ/+^* or *Tbx1^neo2^* to generate heterozygous, WT, null or hypomorphic embryos. We crossed *Tbx1^Cre^* with R26R^mTmG^ mice to map the distribution of *Tbx1-*expressing cells and their descendants. vB12 (cyanocobalamin; Sigma-Aldrich, V2876) was solubilized in PBS and injected intraperitoneally (20 mg/kg). The impact of vB12 on great vessels and ventricular septation defects was scored at E15.5 and E18.5. Pregnant females were injected daily from E7.5 to E11.5. Developmental stages were assessed by considering the morning of vaginal plug as E0.5. Control mice were injected with the same volume of PBS.

Animal studies were carried out under the auspices of the animal protocol 257/2015-PR (licensed to the A.B. laboratory) reviewed, according to Italian regulations, by the Italian Istituto Superiore di Sanità and approved by the Italian Ministero della Salute. All animals are bred into the C57/Bl6J strain for at least five generations. All the mouse lines generated in our laboratory are available through the European public repository EMMA/Infrafrontier.

### Mouse phenotyping

E15.5 and E18.5 embryos were examined under the stereomicroscope and fixed overnight in 4% paraformaldehyde (PFA). E15.5 embryos were embedded in paraffin, sectioned, and stained with Hematoxylin and Eosin. E18.5 hearts and great vessels were manually dissected and photographed under a stereomicroscope, and then embedded in paraffin, sectioned and stained.

### IF

Embryos were fixed overnight in 4% PFA and embedded in wax. For IF analysis, embedded embryos were cut into 7 µm sections. Sections were deparaffinized in xylene, rehydrated, and, after antigen unmasking with citrate buffer, sections were incubated overnight at room temperature with primary antibodies (in 0.5% milk, 10% fetal bovine serum, 1% bovine serum albumin in H_2_O). Each experiment was repeated at least three times.

For antibodies raised in the same species (Fig. 5), we performed two rounds of IF on the same sections. After the first round, using the method described above, we acquired images of the sections and then stripped the antibody by boiling the slides in citrate buffer. We checked the absence of any residual signal and then performed a subsequent incubation with another antibody, before fluorescence detection. We then collected images of the same sections. The two sets of images were compared and merged to identify the overlapping signals. The stripping was considered ineffective if the signals were 100% overlapping, in which case samples were excluded from analysis.

We used the following primary antibodies: anti-GFP (Abcam, ab13970, diluted 1:1000), anti-SNAI2 (Cell Signaling Technology, 9585, diluted 1:100); anti-TFAP2A (Hybridoma Bank, clone 3B5, diluted 1:300); anti-ISL1, (Hybridoma Bank, clone 39.4D5, diluted 1:100); anti-CRE (Millipore, 69050-3, diluted 1:1000); anti-TBX1 (Abcam, ab18530, diluted 1:100).

### Image acquisition

Most images were acquired using a Nikon A1 laser scanning confocal microscope with objective PLANAPO 10×. For SNAI2 quantitative analysis, the images were acquired using a Nikon Motorized Optical Microscope with 10× and 20× objectives. Digital images were saved using the acquisition software provided with the instruments. Microphotographs obtained from the microscopes were cut and labeled to build figures without further processing using Adobe Photoshop.

### Quantitative analysis of IF staining

For SNAI2^+^ cell density analysis, the following numbers of embryos were evaluated: WT E9.5, *n*=5 embryos; *Tbx1*^+/−^ E9.5, *n*=5 embryos; *Tbx1*^−/−^ E9.5, *n*=5 embryos, *Tbx1*^+/−^ B12-treated E9.5, *n*=4 embryos. Cell counts were performed on subsequent transverse sections spanning the entire region of interest (1st PA). Embryos in which immunostaining failed, or sections were damaged, were excluded from the analysis. The region of interest was divided into two segments along the anterior–posterior axis. We counted all the sections from the tag of ruptured buccopharyngeal membrane until the early invagination of the first pharyngeal pouch (8-10 sections for each genotype, anatomically matched). This portion has been identified as A segment. All the sections below the junction, until the end of the arch, were considered as P segment and were entirely analyzed (four to seven sections for each genotype, anatomically matched).

Density distribution heat maps were generated by counting SNAI2^+^ cells in both A and P segments, to record their distribution in the entire structure, on one side (left). Densities were mapped onto a schematic 4×5 grid of equidistant bins placed on the lateral and distal border of the arch and spanning toward the medioproximal portion (Fig. 10D). Bin areas were calculated using the ImageJ software graphic pen by Analyze and Measure tool. The SNAI2^+^ cells were quantified by ImageJ software Cell Counter Pug-in, and the cell density was represented as number of SNAI2^+^ cells/100 µm^2^.

Data from multiple embryos were merged in one schematic arch grid, and a color code was adopted to illustrate the density of SNAI2^+^ cells present in each region (method adapted from Francou et al., 2017). Data were statistically analyzed and graphically represented using Microsoft Office Excel and Prism software. Results were expressed as the mean±s.d. Appropriate statistical tests were used for each sample. Each region of interest was entirely analyzed. No serial sampling was adopted. No randomization or blinding was used. Analysis of data concerning density distribution of SNAI2^+^ cells was performed using the χ2 test followed by unpaired, one-tailed Student's *t*-test. We applied the Benjamini–Hochberg procedure to correct for multiple testing. Statistics on SNAI2^+^ cell numbers were performed by Kruskall–Wallis one-way test, followed by unpaired one-tailed Mann–Whitney test for small samples.

### RNA extraction and RT-PCR

Total RNA was isolated from E9.5 (22 somites) *Tbx1*^+/−^ and *Tbx1*^+/+^ embryos with TRIZOL (Invitrogen) and reverse transcribed using the High Capacity cDNA reverse transcription kit (Applied Biosystem, 4368814).

### RNA-seq gene expression data analysis

For processing and analysis of RNA-seq data, raw data for the high-throughput sequencing of cDNA were generated with Illumina platform for strand-specific paired-end reads. These reads are 125 bp long. In total, eight RNA-seq samples were sequenced. The three biological replicates of RNA-seq for *Tbx1*^+/−^ condition are indicated as Tbx1^+/−^ _rep1, Tbx1^+/−^ _rep2 and Tbx1^+/−^ _rep3. The two biological replicates of RNA-seq for heterozygous with vB12 treatment are denoted as *Tbx1*^+/−^ (+vB12)_rep1 and *Tbx1*^+/−^ (+vB12)__rep2, and the three biological replicates of RNA-seq for WT condition are denoted as Tbx1^+/+^ _rep1, Tbx1^+/+^ _rep2 and Tbx1^+/+^ _rep3. Quality control on raw reads was performed using FastQC (https://www.bioinformatics.babraham.ac.uk/projects/fastqc/).

For alignment of sequence reads, first, the reads were mapped to the mouse genome (mm9) using TopHat2 (version.2.0.7) (Trapnell et al., 2009), with the following options: -G annotation_file.gtf --transcriptome-index transcriptome. All other parameters were used with their default values. The annotation gene transfer format (GTF) file, Mus_musculus.NCBIM37.67.gtf, was downloaded from http://www.ensembl.org.

Gene count matrix was obtained as output using featureCount function from Rsubread R package (version 0.5.4) on ‘exon’ feature type, considering reverse strand for paired end reads with the annotation GTF file. We selected the total counts on 37,620 genes for differential expression analysis.

The raw counts were first filtered by applying the Proportion test, which retained a total of 14488 genes, and then normalized with the upper-quartile method using the RNA-SeqGUI R package (Russo and Angelini, 2014). Principal component analysis (PCA) was performed to separate biological conditions. PCA results showed that samples clustered for different library preparations and different times, and therefore raw data had to be corrected for batch effects. First, we performed a complex design, considering the presence of an unknown batch. We removed batch effects using ARSyNseq function from filtered gene count matrix and considering reads per kilobase of exon per million reads mapped (RPKM) normalization approach. Then, we evaluated differential expression between pair conditions using the non-parametric NOISeqBIO function (Tarazona et al., 2015) after applying upper quartile as normalization method. A posterior probability greater than or equal to 0.95 was used to determine DEGs.

DEGs with absolute value of fold change greater than or equal to 1.2 were considered for pathway analysis. In addition, we performed pathway analysis using the g:Profiler tool (Raudvere et al., 2019), setting the organism to *Mus musculus*, choosing as custom background the list of 14,488 expressed genes in our system, setting the significance threshold for the multiplicity correction ‘fdr’ [i.e. Benjamini and Hochberg false discovery rate (FDR)] with the user threshold 0.05. We limited the sources to GO, Kyoto Encyclopedia of Genes and Genomes (KEGG) and Human Phenotype Ontology databases to evaluate functional enrichment.

To establish the statistical significance of the overlap between sets of genes, we performed the hypergeometric test using the R function phyper.R. Command lines were as follows: Pval.Common=phyper(*q*=467, *m*=3954,*n*=(14488-3954),*k*=1409,lower.tail=FALSE); Pval.1=phyper(*q*=258, *m*=851, *n*=(14488-851),*k*=2092,lower.tail=FALSE) ## Up and down; Pval.2=phyper(*q*=84, *m*=558, *n*=(14488-558),*k*=1862,lower.tail=FALSE) ## down and up; Pval.3=phyper(*q*=58, *m*=851, *n*=(14488-851),*k*=1862,lower.tail=FALSE) ## Up and up; Pval.4=phyper(*q*=64, *m*=558, *n*=(14488-558),*k*=2092,lower.tail=FALSE) ## down and down, where 14,488 is the number of expressed genes, and the other numbers are the different sets.

## Supplementary Material

10.1242/dmm.049415_sup1 Supplementary information
