# Supplementary material for: A phenotypic rescue approach identifies lineage regionalization defects in a mouse model of DiGeorge syndrome
Source: Dis Model Mech. 2022 Sep 27;15(9):dmm049415. doi: 10.1242/dmm.049415 (PMC9555768; doi:10.1242/dmm.049415)
Supplement: Supplementary information [file dmm-15-049415-s1.pdf]

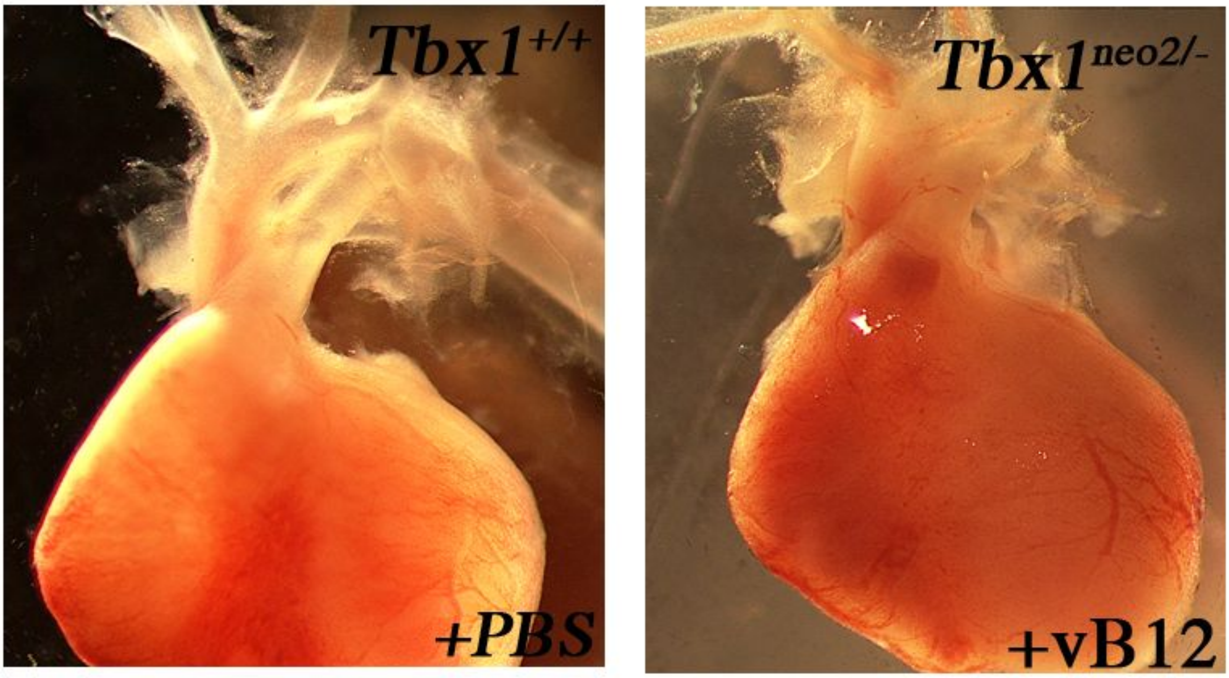

Fig. S1. Whole mount photographs of E18.5 hearts isolated from Tbx1+/+ and Tbx1neo2/- treated with vB12. The two hearts are apparently indistinguishable. Atria have been removed to show the proximal great arteries.

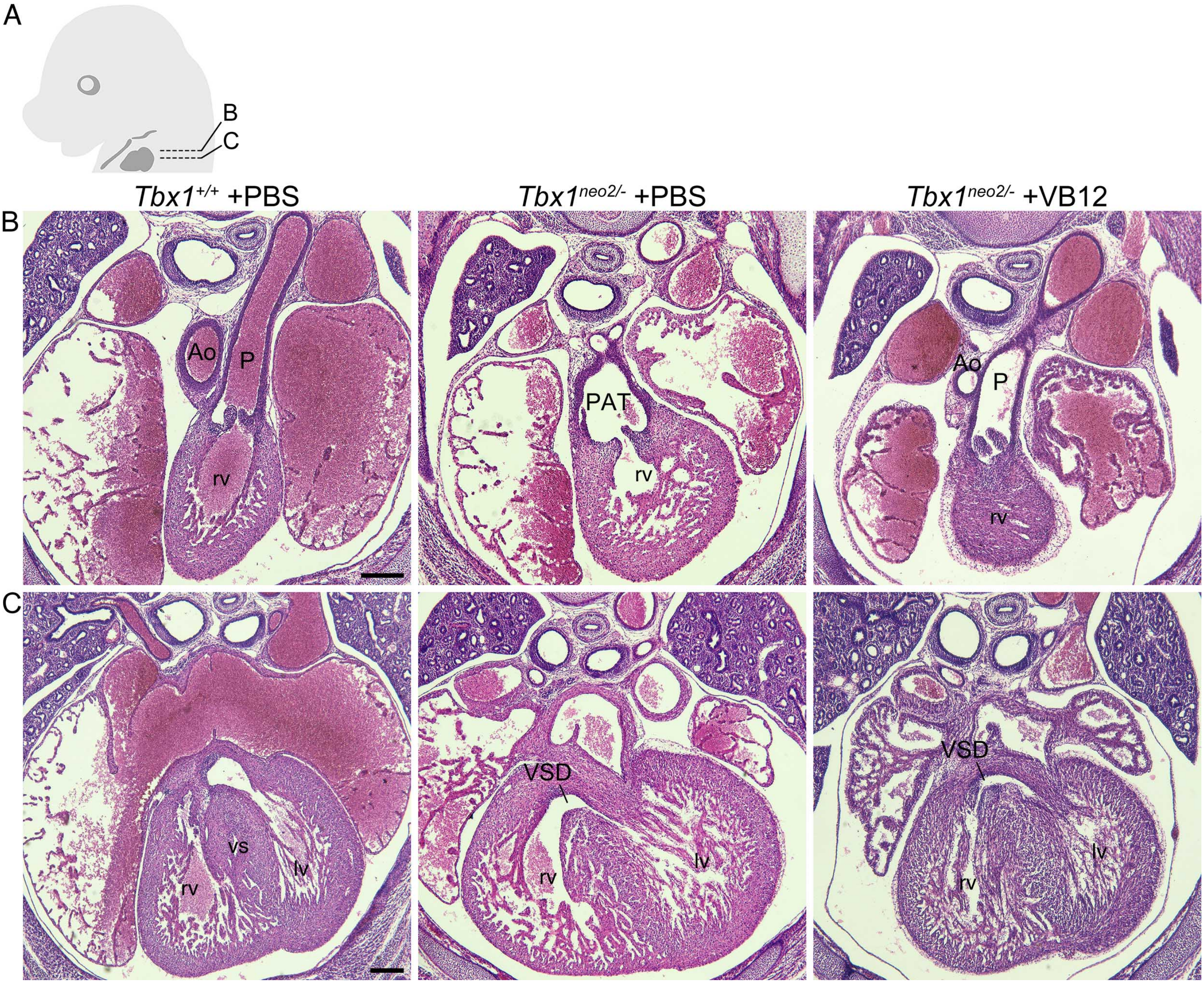

Fig. S2. Vitamin B12 improves cardiac outflow tract defects observed in Tbx1neo2/- mouse embryos at E15.5. A) A diagram showing the section levels. B-C) Transverse histological sections of the heart from Tbx1+/+ and Tbx1neo2/- embryos at E15.5, treated with PBS or vitamin B12. B) Outlet and C) ventricular septal levels. After vB12 treatment, Tbx1neo2/- embryos show separated aorta (Ao) and pulmonary trunk (P), but have overriding aorta (OAo). lv, left ventricle; PTA, Persistent truncus arteriosus; rv, right ventricle; vs, ventricular septum; VSD, ventricular septal defect. Scale bar: 200 µm.

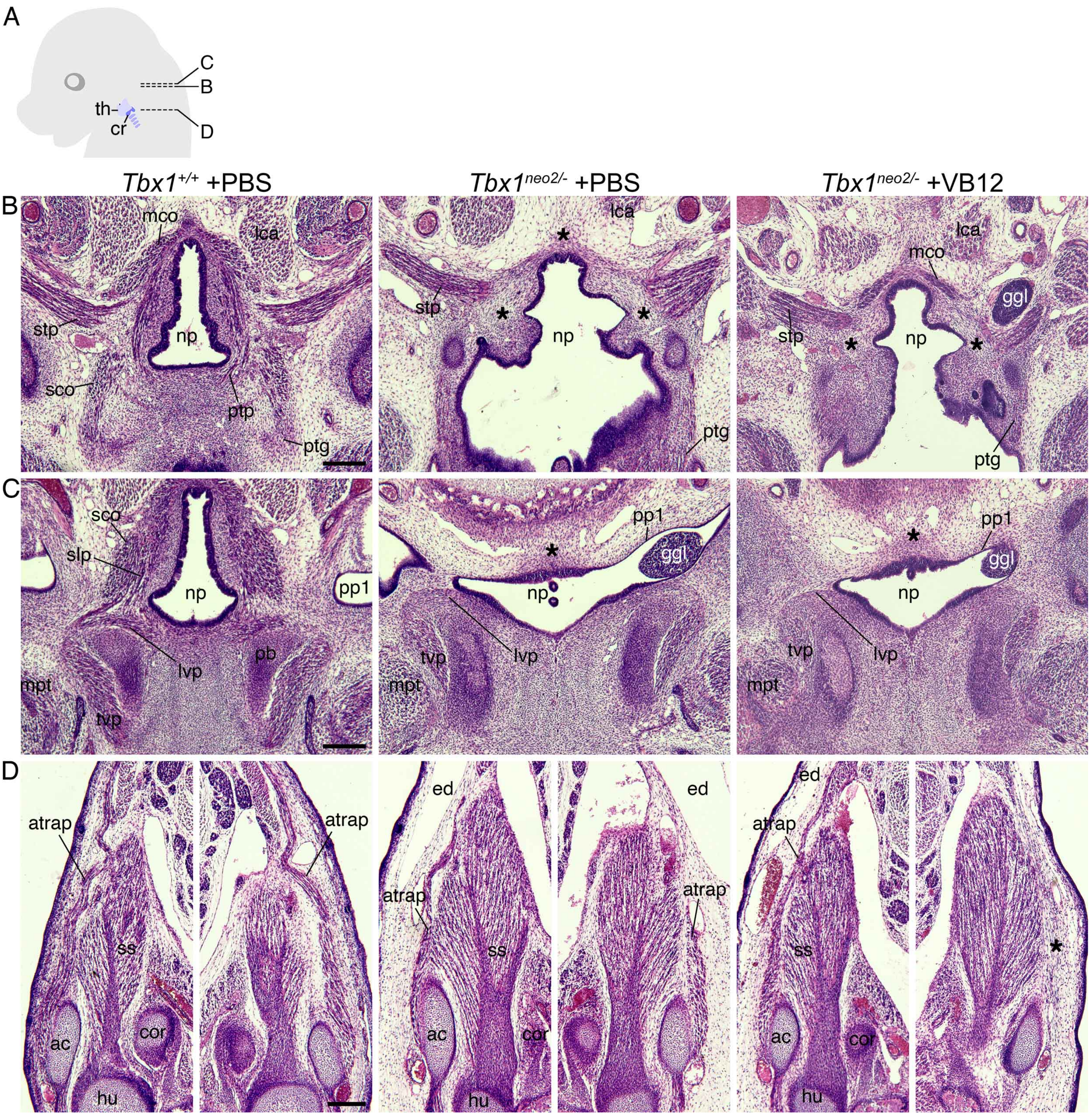

Fig. S3. Vitamin B12 treatment has little effect on the development of branchiomeric muscles derived from 3-6 pharyngeal arches in Tbx1neo2/- embryos. A) A diagram showing the section levels. B-D) Transverse histological sections of Tbx1+/+ and Tbx1neo2/- embryos at E15.5, treated with PBS or vitamin B12. B-C) Nasopharynx and D) shoulder levels. The asterisks indicate missing muscles. Branchiomeric muscles defective in Tbx1neo2/- embryos are not clearly rescued after VB12 treatment. Some muscles exhibit normal development even in the low expression level of Tbx1. ac, acromion; ary, arytenoid cartilage; atrap, acromiotrapezius muscle; cor, coracoid process; ed, edema; ggl, geniculate ganglion-like tissue; hu, humerus; lca, longs capitis muscle; lvp, levator veli palatini muscle; mco, middle constrictor muscle; mpt, medial pterygoid muscle; np, nasopharynx; pb, palatine bone; pp1, first pharyngeal pouch; ptg, palatoglossus muscle; ptp, palatopharyngeus muscle; sco, superior constrictor muscle; slp, salpingopharyngeus muscle; ss, supraspinatus muscle; stp, stylopharyngeus muscle; tvp, tensor veli palati muscle. Scale bar: 200 μm.

Disease Models & Mechanisms • Supplementary information

A

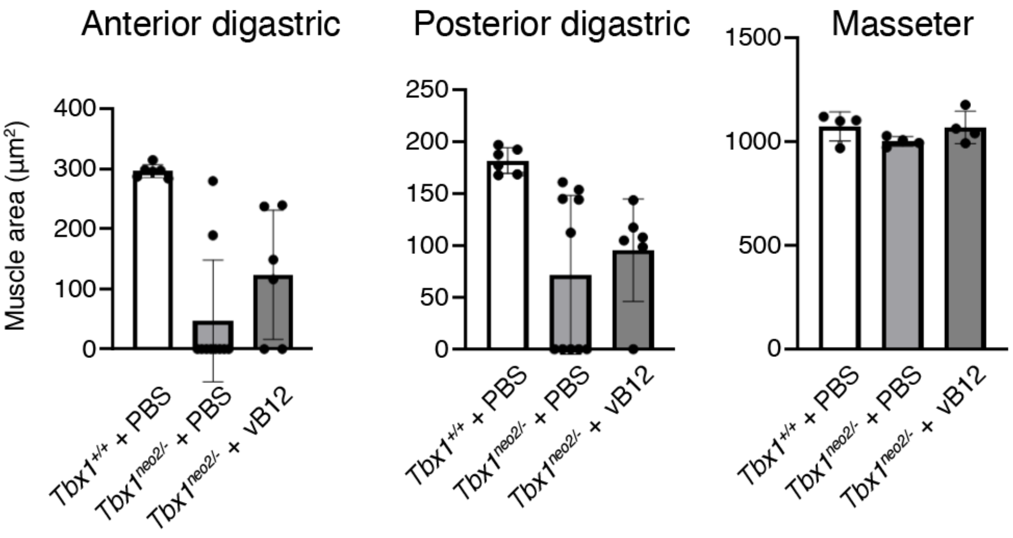

B

PBS- treated *Tbx1neo2/-* (n=5 embryos)							
	P	AU	AB	P'	A	**P'**	**A**
Anterior Digastric	0	2	3	2	8	**7**	**13**
Posterior Digastric	1	3	1	5	5		
Masseter	5	0	0	10	0		
**vB12-treated *Tbx1neo2/-* (n=3 embryos)**							
Anterior Digastric	2	0	1	4	2	**9**	**3**
Posterior Digastric	2	1	0	5	1		
Masseter	3	0	0	6	0	P < 0.05	

Fig. S4. Measurements of selected branchiomeric muscles. A) The histograms show staining area measurements of the indicated muscles. For absent muscles, the value has been set to zero. B) Score of present and absent muscles in the two groups of embryos. Overall, vB12 treatment rescues the formation a significant number of muscles (P<0.05, Chi-squared test). P: Number of embryos with left and right muscles present; AU: Number of embryos with muscle unilaterally absent; AB: Number of embryos with muscles bilaterally absent; P’: Number of muscles present; A: Number of muscles absent.

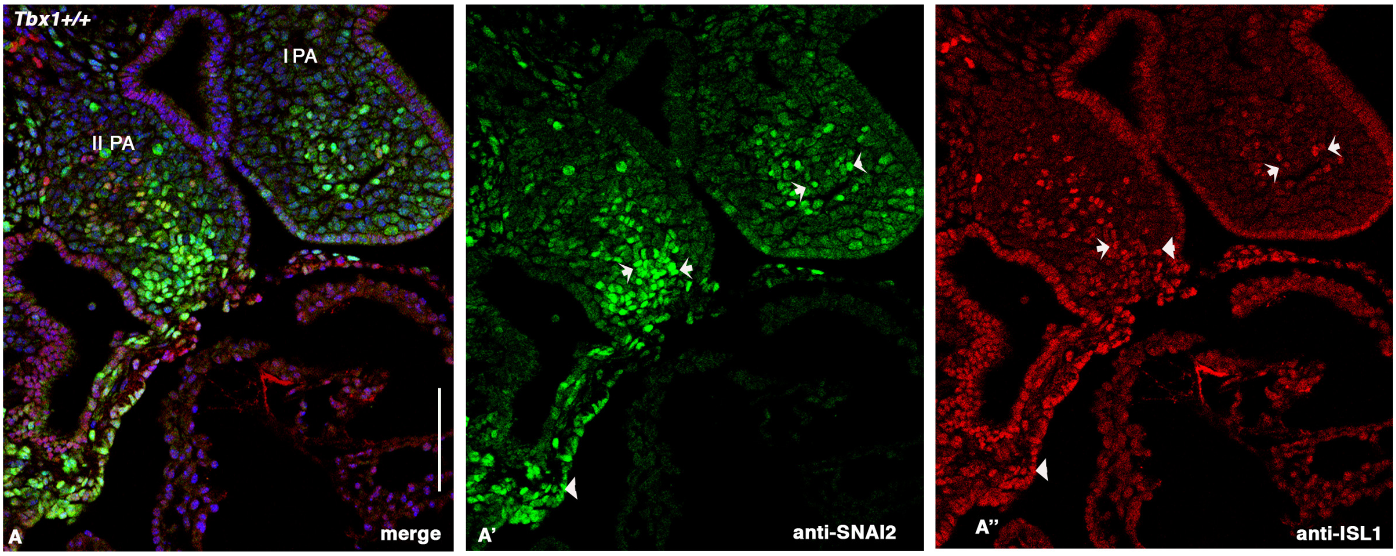

Fig. S5. Sagittal section of a E9.5 embryo double-stained with anti-SNAI2 (panel A', green) and anti-ISL1 (panel A", red). Panel A shows the merged image. Arrows indicate examples of double-stained cells. I PA: 1st Pharyngeal arch; IIP PA: 2nd pharyngeal arch. This is a higher magnification image of a section adjacent to the one shown in Fig. 6A. Scale bar is 200µm.

Disease Models & Mechanisms • Supplementary information

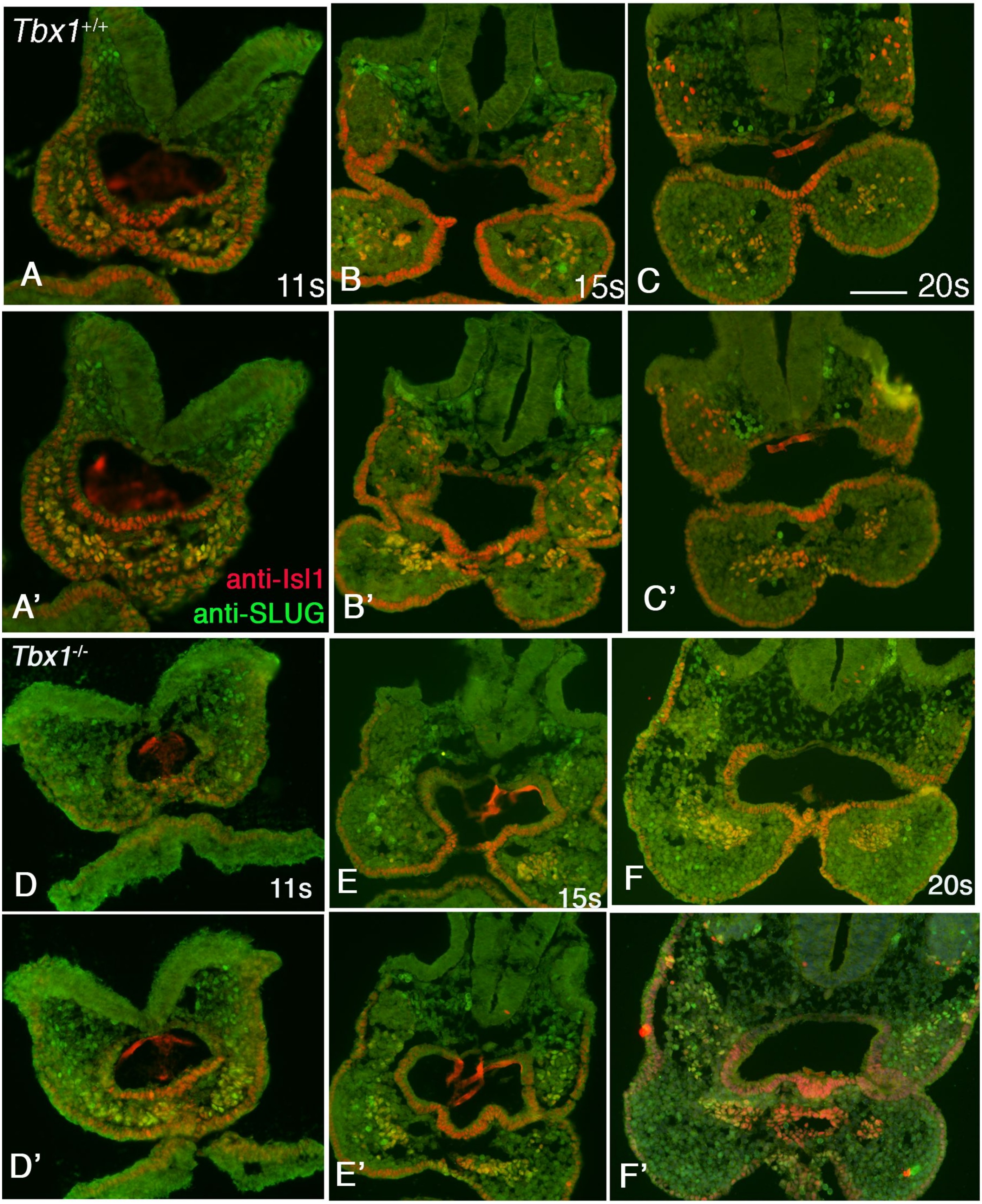

Disease Models & Mechanisms • Supplementary information

Fig. S6. SLUG+ and ISL1+ cells of the 1st pharyngeal arch of Tbx1-/- embryos fail to intermingle with the arch mesenchyme. Immunofluorescence analysis of SLUG and ISL1 at 11, 15 and 22 somites stages of Tbx1+/+ (A-C) and Tbx1-/- (D-F) embryos. Representative images of two consecutive sections of most cranial part of the embryos, at the level of the 1st and 2nd pharyngeal arches. Scale bar: 100 μm

Disease Models & Mechanisms • Supplementary information

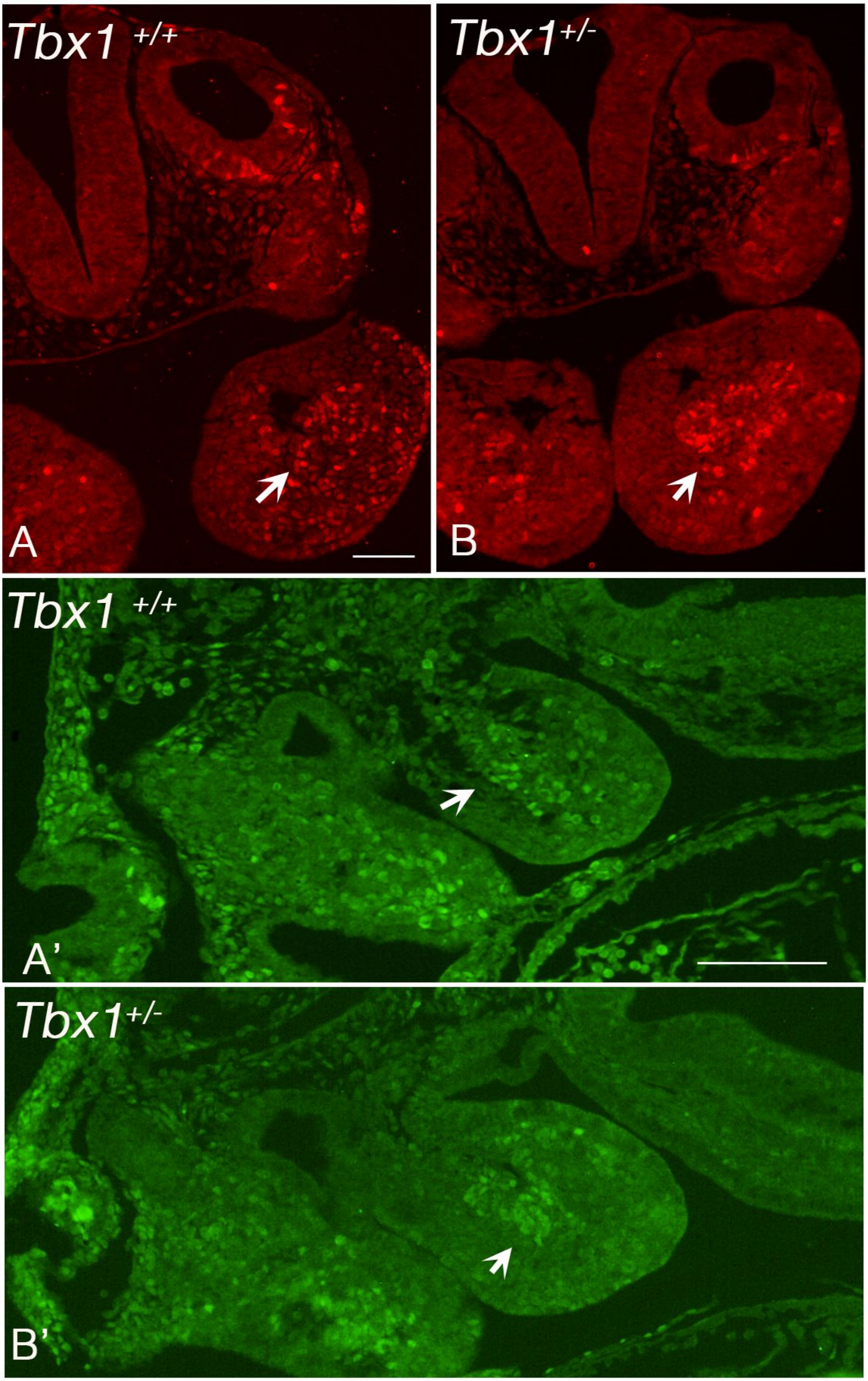

Disease Models & Mechanisms • Supplementary information

Fig. S7. Increased mesodermal cell condensation in the 1st pharyngeal arch of Tbx1 heterozygous embryos at E9.5. Immunofluorescence of transverse (top panels, in red) and sagittal lateral (bottom panels in green) using anti-SLUG antibodies. Arrows point to a group of SLUG+ cells in the 1st pharyngeal arch. Note the higher condensation of SLUG+ cells in the heterozygous mutant. Scale bar: 100 μm.

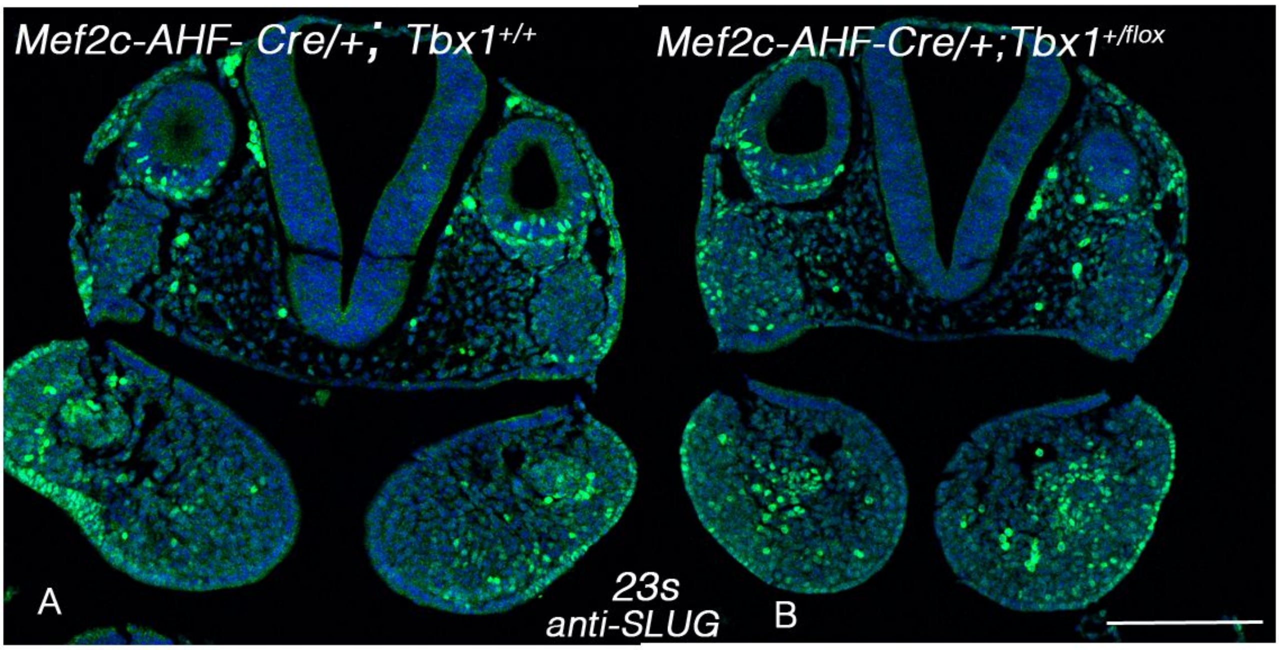

Fig. S8. The condensation phenotype is detectable in conditional mutants. Anti-SLUG immunofluorescence of transverse sections through the 1st pharyngeal arch of E9.5 embryos. The condensation of SLUG+ cells in the core of the arch is also evident in the Mef2c-AHF-Cre;Tbx1flox/+ mutant. Scale bar: 200 µm.

Disease Models & Mechanisms • Supplementary information

Table S1. Banchiomeric muscle phenotype

Branchiomeric muscles		n	Treatment	Normal	Unilateral defect	Bilateral defect
***Tbx1**$^{neo2/-}$* **1st PA muscle**	Ma, Mh, Pt, Te, Tt, Tvp	5	PBS	5	0	0
	Ma, Mh, Pt, Te, Tt, Tvp	3	VB12	3	0	0
	Ad	5	PBS	0	2	3
	Ad	3	VB12	1	1	1
***Tbx1**$^{neo2/-}$* **2nd PA muscle**	Fe	5	PBS	5	0	0
	Fe	3	VB12	3	0	0
	Stm	5	PBS	1	1	3
	Stm	3	VB12	1	2	0
	Pd	5	PBS	1	3	1
	Pd	3	VB12	1	2	0
	Sty	5	PBS	0	0	5
	Sty	3	VB12	0	0	3
***Tbx1**$^{neo2/-}$* **3rd PA muscle**	Stp	5	PBS	5	0	0
	Stp	3	VB12	3	0	0
***Tbx1**$^{neo2/-}$* **4-6th PA muscle**	Crth, Ic, Lvp, Ptg	5	PBS	5	0	0
	Crth, Ic, Lvp, Ptg	3	VB12	3	0	0
	Thar	5	PBS	4	0	1
	Thar	3	VB12	3	0	0
	Otary	5	PBS	1	1	3
	Otary	3	VB12	2	0	1
	Lcary, Mc, Pcary	5	PBS	0	1	4
	Lcary, Mc, Pcary	3	VB12	0	0	3
	Scm	5	PBS	0	2	3
	Scm	3	VB12	0	0	3
	Ptp, Slp, Sc, Trap, Vm	5	PBS	0	0	5
	Ptp, Slp, Sc, Trap, Vm	3	VB12	0	0	3

Ad, anterior digastric muscle; Crth, cricothyroid muscle; Fe, facial expression muscle; Ic, inferior constrictor muscle; Lcary, lateral cricoarytenoid muscle; Lvp, levator veli palatini muscle; Ma, masseter muscle; Mc, middle constrictor muscle; Mh, mylohyoid muscle; Otary, oblique and transverse arytenoid muscle; Pcary, posterior cricoarytenoid muscle; Pd, posterior digastric muscle; Pt, pterygoid muscle; Ptg, palatoglossus muscle; Ptp, palatopharyngeus muscle; Sc, superior constrictor muscle; Scm, sternocleidomastoid muscle; Slp, salpingopharyngeus muscle; Stm, stapedius muscle; Stp, stylopharyngeus muscle; Sty, stylohyoid muscle; Te, temporal muscle; Thary, thyroarytenoid muscle; trap, trapezius muscle; Tt, tensor tympani muscle; Tvp, tensor veli palati muscle; Vm, vocal muscle.

Table S2. Intersection of DEGs *Tbx1*$^{+/-}$ (+vB12) vs *Tbx1*$^{+/-}$(PBS) and *Tbx1*$^{+/-}$ (PBS) vs *Tbx1*$^{+/+}$(PBS)

Click here to download Table S2

Table S3. Gene ontology: *Tbx1*$^{+/-}$ vs WT downregulated genes

Click here to download Table S3
